# Supplementary material for: Genome-Wide Identification and Expression Profiling of Glycosidases, Lipases, and Proteases from Invasive Asian Palm Weevil, Rhynchophorus ferrugineus
Source: Insects. 2025 Apr 17;16(4):421. doi: 10.3390/insects16040421 (PMC12027728; doi:10.3390/insects16040421)
Supplement: Supplementary file 1 [file insects-16-00421-s001.zip › 080425_Supplementary Figures and Tables/Figure S5 - RferLip Allelic variant.pdf]

Figure S5. Allelic variants of RferLip4, 5, 10, 13, 30, 34, 46, 61, 68 and 82 in the locus tags "GWI33_013879, GWI33_016995, GWI33_016501, GWI33_019288, GWI33_001553, GWI33_005342, GWI33_004347, GWI33_007192, GWI33_013879 and GWI33_003404", respectively, and predicted deduced amino acids (with NCBI acc nos). Dots denote identical amino acid residues.

```
                           10        20        30        40        50        60        70        80        90       100
                   ....|....|....|....|....|....|....|....|....|....|....|....|....|....|....|....|....|....|....|....|
RferLip4_KAF7273415  -----------MLQAGFDVLRSFLNTDVPSNKVLETRSDTFAVREIFMREDGLVLYKPQKKTNNSTSRENYEIVLHRPTTETLIQTFSLVRTNTLDEAKA
RferLip4_KAF7273414  MDNFLCTLIMGLVP.KHI..................................................................................

                          110       120       130       140       150       160       170       180       190       200
                   ....|....|....|....|....|....|....|....|....|....|....|....|....|....|....|....|....|....|....|....|
RferLip4_KAF7273415  KFTILHDKLPVFVEIAKEMCNVNGLQKICDVLSEHQSWTVAHLAAYFALYDSFDNAKINCFLNSSDNEKGMSPLQVAITTHNLKTVQILVAANCSLEHLD
RferLip4_KAF7273414  ....................................................................................................

                          210       220       230       240       250       260       270       280       290       300
                   ....|....|....|....|....|....|....|....|....|....|....|....|....|....|....|....|....|....|....|....|
RferLip4_KAF7273415  WDGNSVFHYAASTTKEIIAVLTQGCHPKCLSSRNQNGYTPLHMACLADKPECVKALLIAGADVNKTATGDGDYSEPGYVQNFLLDHPNTLYQNDMKHGGT
RferLip4_KAF7273414  ....................................................................................................

                          310       320       330       340       350       360       370       380       390       400
                   ....|....|....|....|....|....|....|....|....|....|....|....|....|....|....|....|....|....|....|....|
RferLip4_KAF7273415  PLHWANSRQVIDALLDVNCNINAVNFEHKTALHVMVERNRLNCVVALLSRQAKINLPDKNGNHPIHLAVTCGFVNILQALIVFGADLDALNYAGESPRHL
RferLip4_KAF7273414  ....................................................................................................

                          410       420       430       440       450       460       470       480       490       500
                   ....|....|....|....|....|....|....|....|....|....|....|....|....|....|....|....|....|....|....|....|
RferLip4_KAF7273415  AKNQSSKLLYYLHAVGAKRCPPGVAECHEGCSHDGTFDGIIPAKVIGPTNRDTLNQMLAVAGMEVAAKKYKKNDFPKKGRLLCLDGGGIRGLVLVQMLLE
RferLip4_KAF7273414  ....................................................................................................

                          510       520       530       540       550       560       570       580       590       600
                   ....|....|....|....|....|....|....|....|....|....|....|....|....|....|....|....|....|....|....|....|
RferLip4_KAF7273415  LETVFQTPINLCFDWLAGTSTGGILALGIASGKSMKECQSLYFRLKEQTFIGSRPYCSENLENVMKETFGSETVMSDIKHPKVMITGLLADRKPAELHLF
RferLip4_KAF7273414  ....................................................................................................

                          610       620       630       640       650       660       670       680       690       700
                   ....|....|....|....|....|....|....|....|....|....|....|....|....|....|....|....|....|....|....|....|
RferLip4_KAF7273415  RNYTSANDILGVKHDSPYELPPPPQQQYMWQVARATGAAPTYYRMFQRYLDGGLIANNPTLDALTEIQEHCLALKAMNRDDEACPVSVVVSLGTGVIPVT
RferLip4_KAF7273414  ....................................................................................................

                          710       720       730       740       750       760       770       780       790       800
                   ....|....|....|....|....|....|....|....|....|....|....|....|....|....|....|....|....|....|....|....|
RferLip4_KAF7273415  ELKAVDVYRPESIWDTTKVFFGLQNLGTLLVDQATASDGRVVDRGRAWCSSVGIPYFRFSPQMSLEVAMDEKSDDILCNMLWETKAYMHSNMGLMRELAD
RferLip4_KAF7273414  ....................................................................................................


                   ....|
RferLip4_KAF7273415  LLLRG
RferLip4_KAF7273414  .....

                           10        20        30        40        50        60        70        80        90       100
                   ....|....|....|....|....|....|....|....|....|....|....|....|....|....|....|....|....|....|....|....|
RferLip5_KAF7270006  MKIVYLVFTVSVFLVVEISLAGIIDVNWSRSDKLGIEVNLNLPWLPFENETRCYDELGCLNITRSWYHLINRPFNVFPLPRSVINTRFILYTRLNPFEGQ
RferLip5_KAF7270007  ....................................................................................................

                          110       120       130       140       150       160       170       180       190       200
                   ....|....|....|....|....|....|....|....|....|....|....|....|....|....|....|....|....|....|....|....|
RferLip5_KAF7270006  LIKAKNDSIERSNIDVEKKTKFIIHGFIDTPLSNWVKDMRDELLKAEDLNVVVVDWAGGSLPLYTQATANTRLVGLELGYLINYLVKNYEMDPKNVHIIG
RferLip5_KAF7270007  ....................................................................................................

                          210       220       230       240       250       260       270       280       290       300
                   ....|....|....|....|....|....|....|....|....|....|....|....|....|....|....|....|....|....|....|....|
RferLip5_KAF7270006  HSLGAHTAGYAGALVPGLARITGLDPAEPYFQGMPSHVRLDPSDADLVDVIHTDGKGIIFLGMHTPFFAGYGMSQPCGHLDFYPNDGKEQPGCDITQTPL
RferLip5_KAF7270007  ..............................................................--------..............................

                          310       320       330       340       350       360       370       380       390       400
                   ....|....|....|....|....|....|....|....|....|....|....|....|....|....|....|....|....|....|....|....|
RferLip5_KAF7270006  VPLTLIRDGLEEASRVLVACNHVRAIKLFIDSINSNCPYIGRQCSSFKDFMSGKCFGCKSGMSCAVMGYKADTSLGFLENEVQPQERLNEVVGNKYFLTT
RferLip5_KAF7270007  ....................................................................................................

                          410       420       430       440       450       460       470       480       490       500
                   ....|....|....|....|....|....|....|....|....|....|....|....|....|....|....|....|....|....|....|....|
RferLip5_KAF7270006  GKQYPFCQQVYRVSVELAKPTYAEDWVQGRIKASLYSPNGVIKTDLTPAETKLEHGTTYTTVVANPVDLAGNVRKVELSWVYDMNVLEPKSLCLFWCNDH
RferLip5_KAF7270007  ....................................................................................................

                          510       520       530       540       550       560
                   ....|....|....|....|....|....|....|....|....|....|....|....|.
RferLip5_KAF7270006  LYVKSVMVDQLEMPGRGKRDAKFTNRLCSKGKKQYVDISNNGRGIFLDSCAISLSDKNDIS
RferLip5_KAF7270007  .............................................................

                            10        20        30        40        50        60        70        80        90       100
                     ....|....|....|....|....|....|....|....|....|....|....|....|....|....|....|....|....|....|....|....|
RferLip10_ KAF7270545  MTDFEKINNTQQTNESTDNKSDNIKILKNLCGKNAEYFAKDSSENGQRLYISFLAIIDTLNVLYPQVLEVESRVSEFDLDESTPGNGYRSFISIFNMSVN
RferLip10_KAF7270546   ....................................................................................................

                           110       120       130       140       150       160       170       180       190       200
                     ....|....|....|....|....|....|....|....|....|....|....|....|....|....|....|....|....|....|....|....|
RferLip10_ KAF7270545  YAIDLNQKILWRKNSILFRKTVLTKELETCSQLMSSLCTCFKHLQTMMLWSKEGNLFIEDKPLEEIRSVCENVNQYCFYGRSLGFQFCDSLKNVLQFIAL
RferLip10_KAF7270546   ....................................................................................................

                           210       220       230       240       250       260       270       280       290       300
                     ....|....|....|....|....|....|....|....|....|....|....|....|....|....|....|....|....|....|....|....|
RferLip10_ KAF7270545  SMAIFSEAYYSKGSLFSKATSSFMTTTKYITDPEQRAKRIVNISQNADVDFCKAFWFLSESELMNQLPSVVSPTVAVCKVINISPEPLHIVVEGKEIEVP
RferLip10_KAF7270546   ....................................................................................................

                           310       320       330       340       350       360       370       380       390       400
                     ....|....|....|....|....|....|....|....|....|....|....|....|....|....|....|....|....|....|....|....|
```

```
RferLip10_ KAF7270545  VPTSYLGPKPIQVRLISYNNRKGMLGTKSYSNLEAPSRGLLIHCHGGGFVAQSSKSHEGYLRDWAKNLNIPIFSIDYSLAPEAPFPRATEEVFYAYCWAL
RferLip10_KAF7270546   ....................................................................................................

                             410       420       430       440       450       460       470       480       490       500
                       ....|....|....|....|....|....|....|....|....|....|....|....|....|....|....|....|....|....|....|....|
RferLip10_ KAF7270545  KNHEFLGSTGERIVMAGDSAGANLLFSVILKCIHTGIPIPHGMFAAYVPTLVKFVPSPARLLCMMDPLLPFGFLMRCLKAYACPSEVKRISNGAIVNFET
RferLip10_KAF7270546   ....................................................................................................

                             510       520       530       540       550       560       570       580       590       600
                       ....|....|....|....|....|....|....|....|....|....|....|....|....|....|....|....|....|....|....|....|
RferLip10_ KAF7270545  DLDSFEEVSESDLAELQAHKSPVSEASDTLTYGSLTSNVDDVNDRDATSAEIEKSQKYMADIVHKYVL---------------------EADAETEDTKV
RferLip10_KAF7270546   ....................................................................GRDYLGSMFSSKYYNSTSSAG...........

                             610       620       630       640       650       660       670       680       690       700
                       ....|....|....|....|....|....|....|....|....|....|....|....|....|....|....|....|....|....|....|....|
RferLip10_ KAF7270545  PQADTESCTSSVDSSFQTKLFGFVSNIKGHFSKMLGEHGGSDMILETDKPNTLWDDLCFPVPNNPYLSPYCASDEDLKKFPPTKILTVQMDPCLDDCVTF
RferLip10_KAF7270546   ....................................................................................................

                             710       720       730       740       750
                       ....|....|....|....|....|....|....|....|....|....|....|....
RferLip10_ KAF7270545  AKKLRWVGTEVHLDILEGLPHGFLNFSLISRDAHEGSKLCMQRMKELLNIHPESIKSNK
RferLip10_KAF7270546   ...........................................................

                             10        20        30        40        50        60        70        80        90        100
                       ....|....|....|....|....|....|....|....|....|....|....|....|....|....|....|....|....|....|....|....|
RferLip13_KAF7267490   MNLSFAGCGFLGIYHVGVACCFRKYAPHLLLNKISGASAGAMAACCLLLDLPLGETTSDVLRVATEARKKSLGPFNPSFNIHQLLLEGLERFLPDDAHIR
RferLip13_KAF7267491   ............M............T.....D......F..I..........................................................

                             110       120       130       140       150       160       170       180       190       200
                       ....|....|....|....|....|....|....|....|....|....|....|....|....|....|....|....|....|....|....|....|
RferLip13_KAF7267490   VSGKLHISLTRVRDGKNVIVSQFDSREELIQALLASAFIPVFSGLFPPKFKGVRYMDGGYSDNLPTLDEHTITVSPFCGESDICPRDDSSQLFHINIANT
RferLip13_KAF7267491   ....................................................................................................

                             210       220       230       240       250       260       270       280       290       300
                       ....|....|....|....|....|....|....|....|....|....|....|....|....|....|....|....|....|....|....|....|
RferLip13_KAF7267490   SIELSKHNIYRMVRILFPPPPETLANMCKQGFDDALRFLHKNNLINCTKCLAVQSTFVVSDTLEENLIYDPQCTDCKIHRQEALVSNVPDTVLNVFQEVI
RferLip13_KAF7267491   ....................................................................................................

                             310       320       330       340       350       360       370       380       390       400
                       ....|....|....|....|....|....|....|....|....|....|....|....|....|....|....|....|....|....|....|....|
RferLip13_KAF7267490   ESANKGVSNWLFKHRGMKLLSVLSLPYTLPADIMYATFTKLHLLKPVVPQLKRRLVSHNATSFQLLTSIGNQFETFSFDYKPCQALLPHNRNACIKDPSL
RferLip13_KAF7267491   ....................................................................................................

                             410       420       430       440       450       460       470       480       490       500
                       ....|....|....|....|....|....|....|....|....|....|....|....|....|....|....|....|....|....|....|....|
RferLip13_KAF7267490   SVHERFMATAPQVGNALWDVSKVVLDQMSNVLTRVNRKRQQISAKITCQLAITEYSGGKEPPVDDLVENKVNLNFTLDLDDCDLPLEQSPERKFNTRQIL
RferLip13_KAF7267491   ....................................................................................................

                             510       520       530       540       550       560       570       580       590       600
                       ....|....|....|....|....|....|....|....|....|....|....|....|....|....|....|....|....|....|....|....|
RferLip13_KAF7267490   QRKPSFTINKSDTMDDDTFDHILQVTSHHEAIMAYYYLDENNKVKVTEIFDVTEDESPLLQTPCEKDVNTNLEFDDSWTDYNQVGELAQDDLHSEYSLED
RferLip13_KAF7267491   ....................................................................................................

                             610       620       630       640       650       660
                       ....|....|....|....|....|....|....|....|....|....|....|....|....|..
RferLip13_KAF7267490   LLDSDKNVFSDPESEWVGTCKVEEPEDDLRYSSNDARPESDRVTLKNEDNTKEKLLIHSRDFEYKNC
RferLip13_KAF7267491   ...................................................................

                             10        20        30        40        50        60        70        80        90        100
                       ....|....|....|....|....|....|....|....|....|....|....|....|....|....|....|....|....|....|....|....|
RferLip30_KAF7283047   MGVFANHNESLNYRYACPDKFTAIGRKCYRFYGGTSTWFQAFYACKNLNDSTLTIFSNRQELLQFESFLVKDKLKTVGQNRTFWVGAFKDWQQKKWIYTD
RferLip30_KAF7283048   ....................................................................................................

                             110       120       130       140       150       160       170       180       190       200
                       ....|....|....|....|....|....|....|....|....|....|....|....|....|....|....|....|....|....|....|....|
RferLip30_KAF7283047   GTPVKYLSLREFSKNGHDNWTCLLVDTQRNRWRSENCMNSIPFVCETEAKMSVEFKTTDKKIDRRSNVHRCVERYNSLTDKQKKKCQKLKKKMAQTNSES
RferLip30_KAF7283048   ....................................................................................................

                             210       220       230       240       250       260       270       280       290       300
                       ....|....|....|....|....|....|....|....|....|....|....|....|....|....|....|....|....|....|....|....|
RferLip30_KAF7283047   ---KTQAAYPSKPTTKGISYLCPQNWMLLGNQCYLFSTKNATWSDAHFNCASINAHLAIIKTKAQDKKLRIYLNQFTERKERWIGGRYNGKTNEWVWALN
RferLip30_KAF7283048   VIQ.................................................................................................

                             310       320       330       340       350       360       370       380       390
                       ....|....|....|....|....|....|....|....|....|....|....|....|....|....|....|....|....|....|.
RferLip30_KAF7283047   ARPLQFKGFAEEVLQNSTIATEWQALIVDPQYSYQWNFRNEMEEHCYICQVKGRMVKKLRQPRGTTIEITSHPRYKIRSTVGGRSKRNKRH
RferLip30_KAF7283048   ...........................................................................................

                             10        20        30        40        50        60        70        80        90        100
                       ....|....|....|....|....|....|....|....|....|....|....|....|....|....|....|....|....|....|....|....|
RferLip34_KAF7286424   MSLIFTFHSFGELVESILALIGSVLLPLLPVVFGILAYFDIISLTFLKVALFFCFVLFVILPIIYKYSYTLQRYAVFLTFVHVPAFPDYKNPSAYGLKGS
RferLip34_KAF7286423   ------------------------------MLM.KRRFIRR...........................................................

                             110       120       130       140       150       160       170       180       190       200
                       ....|....|....|....|....|....|....|....|....|....|....|....|....|....|....|....|....|....|....|....|
RferLip34_KAF7286424   RNFYINTDDGVKLGVWQILPENITDFEGSDESFEDILDNGQDIIIYSHGNGGTRLSDHRIEMYKVLRKFFHVFAFDYRGYGDSSSSSPSEIGCVNDLLNI
RferLip34_KAF7286423   ....................................................................................................

                             210       220       230       240       250       260       270       280       290       300
                       ....|....|....|....|....|....|....|....|....|....|....|....|....|....|....|....|....|....|....|....|
RferLip34_KAF7286424   YKWIQNRTKSLDRPLGLILEAPFNNMKEEISEFPLAQLFKHLPWFKMTVVNPMAKNFPFTTDKYICGIDIPVMILHAKDDKVVPYKLGYKLYQSAEKCRL
RferLip34_KAF7286423   ....................................................................................................

                             310       320       330       340       350
                       ....|....|....|....|....|....|....|....|....|....|..
RferLip34_KAF7286424   DTQGGVIFHSFDEKYHFGHKFICKALDLSDKIRDFTTYVYTFQKNHKTVLNE
RferLip34_KAF7286423   ....................................................

                             10        20        30        40        50        60        70        80        90        100
                       ....|....|....|....|....|....|....|....|....|....|....|....|....|....|....|....|....|....|....|....|
```

```
RferLip46_KAF7286724  MLMLTVCIFAIFIIEIEAVTVIDNENKIFLNRFGYIKNFSMKKVGRRLSTNNSFQFNLDTRDGTRSKRGVLNLYNMVSCATRCNPLSYKGYGCFCGFLGS
RferLip46_KAF7286723  ....................................................................................................

                             110       120       130       140       150       160       170       180       190       200
                      ....|....|....|....|....|....|....|....|....|....|....|....|....|....|....|....|....|....|....|....|
RferLip46_KAF7286724  GTPVDGIDTCCKLHDLCYVDAECPMYLEYFVPYYWRCWNNKPICAFNQGPFENSSPCAERLCECDRALSECFSHFACPSIQPICESSPLRVLKKALMIFL
RferLip46_KAF7286723  ...........................................S-.......................................................


                              10        20        30        40        50        60        70        80        90       100
                      ....|....|....|....|....|....|....|....|....|....|....|....|....|....|....|....|....|....|....|....|
RferLip61_KAF7279478  MPGLIVFRRRWSVGSDDLVVPGAFLFTTHFIWTIILTVILIVVKYDRSVQCVLLLWGLVIGYLVILILSMVIEICVCVVALRGSILDTGPRSSMQYILYI
RferLip61_KAF7279477  ....................................................................................................

                             110       120       130       140       150       160       170       180       190       200
                      ....|....|....|....|....|....|....|....|....|....|....|....|....|....|....|....|....|....|....|....|
RferLip61_KAF7279478  RLSVMIIEAGWLSAGVAWLMNYYVDCPIENAKETVLAMVVFNWCILLSVLITVWCCYDTAGRSWVKMKQYQRSMRESESKFQYKRSGSTRNWRQRKVLRA
RferLip61_KAF7279477  ....................................................................................................

                             210       220       230       240       250       260       270       280       290       300
                      ....|....|....|....|....|....|....|....|....|....|....|....|....|....|....|....|....|....|....|....|
RferLip61_KAF7279478  YQDSWHNRCRFLFCCSTPSDRNRNSFADIARLLSDFFRDLDVVPSDVIVGLVLLRKFQKIERKAIVEQRKNDTYEFLSGVAITPRTQFLSLHEDGNDLEL
RferLip61_KAF7279477  ....................................................................................................

                             310       320       330       340       350       360       370       380       390       400
                      ....|....|....|....|....|....|....|....|....|....|....|....|....|....|....|....|....|....|....|....|
RferLip61_KAF7279478  FQTVIHYAHYAVRAYGWPIHVVMNKTGICHLCTGLQCCCLPCRKHTEHDAEVVDDNCCRCNFAALQKLTNLGDIEIIYATYHVDVGETPFFVAVDYDRKA
RferLip61_KAF7279477  ....................................................................................................

                             410       420       430       440       450       460       470       480       490       500
                      ....|....|....|....|....|....|....|....|....|....|....|....|....|....|....|....|....|....|....|....|
RferLip61_KAF7279478  IVISVRGTLSMKDILTDLNAESETIPLDPPREDWTGHKGMVQAAQYILDKIEQEQLLERARARSPDRGTRDFDVVIVGHSLGAGTASILGILMRQLYPSL
RferLip61_KAF7279477  ....................................................................................................

                             510       520       530       540       550       560       570       580       590       600
                      ....|....|....|....|....|....|....|....|....|....|....|....|....|....|....|....|....|....|....|....|
RferLip61_KAF7279478  QCFCYSPPGGLLSSPAVEYTKEFTVSVVVGKDVVPRIGLHQMETLRTDLINAIKRSVDPKWKTITCSIICCGCSQPTSAVEMSTGESEVSEYMRSKHNAR
RferLip61_KAF7279477  ....................................................................................................

                             610       620       630       640       650       660       670       680       690       700
                      ....|....|....|....|....|....|....|....|....|....|....|....|....|....|....|....|....|....|....|....|
RferLip61_KAF7279478  SMGIHPSDSTIALTSHQPLYPPGRIIHVVRHHPTTG--------QQALSKKEPVYQALWASNTDFDEVLISPVMIQDHMPDKVLEALNKCVTGGQEPTSP
RferLip61_KAF7279477  ....................................QQKYEKRWR...........................................------------

                             710       720       730       740       750       760       770       780       790       800
                      ....|....|....|....|....|....|....|....|....|....|....|....|....|....|....|....|....|....|....|....|
RferLip61_KAF7279478  SAGCRQGRIFGFRSQASTFRYQVVTTTGPKKPHRSSSTTDSTTNYFSASSNPNLFLETSFTSLQSPSSNSSYTNGYYSTKSHPSPVSFRHPASGFNISIR
RferLip61_KAF7279477  ---------------------K..............................................................................

                             810       820       830       840       850       860       870       880       890       900
                      ....|....|....|....|....|....|....|....|....|....|....|....|....|....|....|....|....|....|....|....|
RferLip61_KAF7279478  SNQSDASFKSQQLSLTKHEEPPSITKSLSLNLVPKIDLIHDDWLGLAPLASPESLSELSSISSRTSLVATSVVVDICATPKVMRRTPKIIGSLSTCADDI
RferLip61_KAF7279477  ....................................................................................................

                             910       920       930       940       950       960       970       980       990      1000
                      ....|....|....|....|....|....|....|....|....|....|....|....|....|....|....|....|....|....|....|....|
RferLip61_KAF7279478  RNIRNFEKCRVFPRLIVNNNIESSSSSNLSFESASSNNKCCSTSSRQDAGNSQKNTLEVVSTRDGRTSSGSEFQSAEDILDNVLTSAGCKEFFNSDSNIL
RferLip61_KAF7279477  ....................................................................................................

                            1010      1020      1030      1040      1050      1060      1070      1080      1090      1100
                      ....|....|....|....|....|....|....|....|....|....|....|....|....|....|....|....|....|....|....|....|
RferLip61_KAF7279478  EMHSDLLAEGSPPPGYFGSSPTTPIYPQILSPGSPQELDPDILSNLAIDLSMDDEHRSIQFHPSSKASSSVDSGSVCRTPSDTTKNVTFNPQVITVDTIC
RferLip61_KAF7279477  ....................................................................................................

                            1110      1120      1130      1140      1150      1160      1170      1180      1190      1200
                      ....|....|....|....|....|....|....|....|....|....|....|....|....|....|....|....|....|....|....|....|
RferLip61_KAF7279478  EASYSPPVRWRLFRSRNKKQADLPKLPPIPTTISSAVPIKTILSKGKRTEPEERLSEAAASDEQCFSKTEALPLLSGLSNSASDRTPSPNFVRRKKYVYP
RferLip61_KAF7279477  ....................................................................................................

                            1210
                      ....|....|....|
RferLip61_KAF7279478  SDIVVMSKPPHESAV
RferLip61_KAF7279477  ...............

                              10        20        30        40        50        60        70        80        90       100
                      ....|....|....|....|....|....|....|....|....|....|....|....|....|....|....|....|....|....|....|....|
RferLip68_KAF7273415  -----------MLQAGFDVLRSFLNTDVPSNKVLETRSDTFAVREIFMREDGLVLYKPQKKTNNSTSRENYEIVLHRPTTETLIQTFSLVRTNTLDEAKA
RferLip68_KAF7273414  MDNFLCTLIMGLVP.KHI..................................................................................

                             110       120       130       140       150       160       170       180       190       200
                      ....|....|....|....|....|....|....|....|....|....|....|....|....|....|....|....|....|....|....|....|
RferLip68_KAF7273415  KFTILHDKLPVFVEIAKEMCNVNGLQKICDVLSEHQSWTVAHLAAYFALYDSFDNAKINCFLNSSDNEKGMSPLQVAITTHNLKTVQILVAANCSLEHLD
RferLip68_KAF7273414  ....................................................................................................

                             210       220       230       240       250       260       270       280       290       300
                      ....|....|....|....|....|....|....|....|....|....|....|....|....|....|....|....|....|....|....|....|
RferLip68_KAF7273415  WDGNSVFHYAASTTKEIIAVLTQGCHPKCLSSRNQNGYTPLHMACLADKPECVKALLIAGADVNKTATGDGDYSEPGYVQNFLLDHPNTLYQNDMKHGGT
RferLip68_KAF7273414  ....................................................................................................

                             310       320       330       340       350       360       370       380       390       400
                      ....|....|....|....|....|....|....|....|....|....|....|....|....|....|....|....|....|....|....|....|
RferLip68_KAF7273415  PLHWANSRQVIDALLDVNCNINAVNFEHKTALHVMVERNRLNCVVALLSRQAKINLPDKNGNHPIHLAVTCGFVNILQALIVFGADLDALNYAGESPRHL
RferLip68_KAF7273414  ....................................................................................................

                             410       420       430       440       450       460       470       480       490       500
                      ....|....|....|....|....|....|....|....|....|....|....|....|....|....|....|....|....|....|....|....|
RferLip68_KAF7273415  AKNQSSKLLYYLHAVGAKRCPPGVAECHEGCSHDGTFDGIIPAKVIGPTNRDTLNQMLAVAGMEVAAKKYKKNDFPKKGRLLCLDGGGIRGLVLVQMLLE
RferLip68_KAF7273414  ....................................................................................................

                             510       520       530       540       550       560       570       580       590       600
```

```
                     ....|....|....|....|....|....|....|....|....|....|....|....|....|....|....|....|....|....|....|....|
RferLip68_KAF7273415  LETVFQTPINLCFDWLAGTSTGGILALGIASGKSMKECQSLYFRLKEQTFIGSRPYCSENLENVMKETFGSETVMSDIKHPKVMITGLLADRKPAELHLF
RferLip68_KAF7273414  ....................................................................................................

                           610       620       630       640       650       660       670       680       690       700
                     ....|....|....|....|....|....|....|....|....|....|....|....|....|....|....|....|....|....|....|....|
RferLip68_KAF7273415  RNYTSANDILGVKHDSPYELPPPPQQQYMWQVARATGAAPTYYRMFQRYLDGGLIANNPTLDALTEIQEHCLALKAMNRDDEACPVSVVVSLGTGVIPVT
RferLip68_KAF7273414  ....................................................................................................

                           710       720       730       740       750       760       770       780       790       800
                     ....|....|....|....|....|....|....|....|....|....|....|....|....|....|....|....|....|....|....|....|
RferLip68_KAF7273415  ELKAVDVYRPESIWDTTKVFFGLQNLGTLLVDQATASDGRVVDRGRAWCSSVGIPYFRFSPQMSLEVAMDEKSDDILCNMLWETKAYMHSNMGLMRELAD
RferLip68_KAF7273414  ....................................................................................................


                     ....|
RferLip68_KAF7273415  LLLRG
RferLip68_KAF7273414  .....

                           10        20        30        40        50        60        70        80        90        100
                     ....|....|....|....|....|....|....|....|....|....|....|....|....|....|....|....|....|....|....|....|
RferLip82_KAF7287772  --MKFL------------------------------CSLGLLILSLDFCC--CFIVQR-------------SENVTCSKNEFRCGNGKCIPGHWQCDNDI
RferLip82_KAF7287770  MLLR.PNVTFVFIGILCYFFGGSIGNPGSNATEGSQ...RQFRCANKK.IPVA.VCDNENDCGDNSDELAC.K...........................

                           110       120       130       140       150       160       170       180       190       200
                     ....|....|....|....|....|....|....|....|....|....|....|....|....|....|....|....|....|....|....|....|
RferLip82_KAF7287772  DCSDESDENEHICQQKKCGADEFTCRSAPGECVPLTWMCDDNQDCSDGSDEKSCNETCRADEFTCKNGKCIQQKWVCDADNDCGDNSDEVDCPAVSCAPE
RferLip82_KAF7287770  ....................................................................................................

                           210       220       230       240       250       260       270       280       290       300
                     ....|....|....|....|....|....|....|....|....|....|....|....|....|....|....|....|....|....|....|....|
RferLip82_KAF7287772  TEFQCSERFCVPSKWHCDGEYDCMNGRDEQGCPPRPHSSVCLPSEHECNDLITCIHKGWLCDGTKDCPDGSDEEPAHCSNITCRADQFQCADRSCIAGPL
RferLip82_KAF7287770  ....................................................................................................

                           310       320       330       340       350       360       370       380       390       400
                     ....|....|....|....|....|....|....|....|....|....|....|....|....|....|....|....|....|....|....|....|
RferLip82_KAF7287772  LCDGQPNCPDGSDERDCGKPAAICDPITHFSCGPGGPCVPLSHVCDGKPDCPAWEDEPRGSCGINECAKDNGGCAHRCIDTPAGFRCECRMGYALTHDNR
RferLip82_KAF7287770  ....................................................................................................

                           410       420       430       440       450       460       470       480       490       500
                     ....|....|....|....|....|....|....|....|....|....|....|....|....|....|....|....|....|....|....|....|
RferLip82_KAF7287772  TCRDIDECQIEGSCSQICRNDKGHASLLFARRRDIRKISLDHHEMTSIVNETNSATALDFVFRTGMIFWSDVADKKIYKAPIDEGNAKTVVVSDEVTTSD
RferLip82_KAF7287770  ....................................................................................................

                           510       520       530       540       550       560       570       580       590       600
                     ....|....|....|....|....|....|....|....|....|....|....|....|....|....|....|....|....|....|....|....|
RferLip82_KAF7287772  GLAVDWVYEHLYWTDTGTNTISLANFDGQMRKVLIRDDLEEPRAIAVDPLEGWMFWTDWGQEPRIERAGMDGSHRQAIVTYDVRWPNGLTLDLVKKRLYW
RferLip82_KAF7287770  ....................................................................................................

                           610       620       630       640       650       660       670       680       690       700
                     ....|....|....|....|....|....|....|....|....|....|....|....|....|....|....|....|....|....|....|....|
RferLip82_KAF7287772  VDAKLNTISACDWDGQNRKLILFSETALRHPFSITTFEDWLYWTDWDRAAVFKANKFTGYDLAPITATEMVQNPMVIHVYHPYRQPDAENHCQPVNGHCS
RferLip82_KAF7287770  ....................................................................................................

                           710       720       730       740       750       760       770       780       790       800
                     ....|....|....|....|....|....|....|....|....|....|....|....|....|....|....|....|....|....|....|....|
RferLip82_KAF7287772  HLCLPAPRIGLRAPSISCACPEGLRMLSDGLTCVQDESITTSTPSQSEPYSPRAGPIPESTTNRAKNGPGTSIPESLDNGGVAFAVMFGIVVILFALGII
RferLip82_KAF7287770  ....................................................................................................

                           810       820       830       840       850       860
                     ....|....|....|....|....|....|....|....|....|....|....|....|..
RferLip82_KAF7287772  ARLGYNRLKQRNVTSMNFDNPVYRKTTEDQFTLEKNNFPVKPYLSTVGEEAQQPLTNNHDPV
RferLip82_KAF7287770  ..............................................................
```
