# Supplementary material for: Genome-Wide Identification and Expression Profiling of Glycosidases, Lipases, and Proteases from Invasive Asian Palm Weevil, Rhynchophorus ferrugineus
Source: Insects. 2025 Apr 17;16(4):421. doi: 10.3390/insects16040421 (PMC12027728; doi:10.3390/insects16040421)
Supplement: Supplementary file 1 [file insects-16-00421-s001.zip › 080425_Supplementary Figures and Tables/Figure S6 - RferPro Allelic variant.pdf]

Figure S6. Allelic variants of RferPro4, 10, 16, 30, 38, 42, 115, 117, 132, 140, 177, 193, 80, 105, 120, 123, 128, 33, 56, 70 and 119 and predicted deduced amino acids (with NCBI acc nos). Dots denote identical amino acid residues. Locus_tag identifiers and scaffold position numbers are included in the Table S9.

```
                            10        20        30        40        50        60        70        80        90       100
                    ....|....|....|....|....|....|....|....|....|....|....|....|....|....|....|....|....|....|....|....|
RferPro4_KAF7265590 MTKKYKYDYSNPFNLDDMDSEKDSIHSVPFWMGLLIILCLGLLFGFVFLIGTLLPTPLTVNDEANYPDSFIADRAAQDLRYLTDLGPKITGSFENEYAVV
RferPro4_KAF7265591 ....................................................................................................

                           110       120       130       140       150       160       170       180       190       200
                    ....|....|....|....|....|....|....|....|....|....|....|....|....|....|....|....|....|....|....|....|
RferPro4_KAF7265590 EYLSLHIKTIISEANPNQYIEMDLQEATGSYYLDDSIGFHNIYHKVQNVVVKIHGESNNTKQSILLNSHFDTVPSSPGASDDGINVVTMLEIIRKLSQRE
RferPro4_KAF7265591 ....................................................................................................

                           210       220       230       240       250       260       270       280       290       300
                    ....|....|....|....|....|....|....|....|....|....|....|....|....|....|....|....|....|....|....|....|
RferPro4_KAF7265590 QRLNQNIIFLFNGAEETPLQAAHGFVAHHKWAEESKYLINLEACGSGGKIILFQTGPDAPWLVKYYSTVPHPYGNVIGEEVFQSGLIPSDTDFRLFRDFG
RferPro4_KAF7265591 ....................................................................................................

                           310       320       330       340       350       360       370       380       390       400
                    ....|....|....|....|....|....|....|....|....|....|....|....|....|....|....|....|....|....|....|....|
RferPro4_KAF7265590 NMVGADFAFADDGYRYHTEYDSFENIPLGSYQHVGDNILDFVIKIGNALETTEKERSLGKAIYFDIFGLFMITYSSKTAVILNVCIALVSILVFFLSIVI
RferPro4_KAF7265591 ....................................................................................................

                           410       420       430       440       450       460       470       480       490       500
                    ....|....|....|....|....|....|....|....|....|....|....|....|....|....|....|....|....|....|....|....|
RferPro4_KAF7265590 FRVRINVETLRYLSITTAAIVGSWLLAGIFVMILAVVLDCLGKTMSWYANPWTTFGLYIIPVIGISGCLPYFTKYK-----NFSLNIRGQFQAHLVRLIW
RferPro4_KAF7265591 ............................................................................FIYFQ...................

                           510       520       530       540       550       560       570       580       590       600
                    ....|....|....|....|....|....|....|....|....|....|....|....|....|....|....|....|....|....|....|....|
RferPro4_KAF7265590 TFVLIIGTAAQVRSTYFIMILILFDSFGFLLIHALRLQYTVGIWLIIHVLSLVLPVIYMMTVAAMNFNFFIPITGRLGSQLNPELLIAASALFFTIVITS
RferPro4_KAF7265591 ....................................................................................................

                           610       620       630       640       650       660       670       680       690       700
                    ....|....|....|....|....|....|....|....|....|....|....|....|....|....|....|....|....|....|....|....|
RferPro4_KAF7265590 PLTVLTNMLRKAIWYYLTLAVIFATFIGLLYTPFAFPYSADQSSPKPQRHWMMHTQRQFFLESGELSRTESGFFMLDMDRNSPSILKRYVKELADVESLQ
RferPro4_KAF7265591 ....................................................................................................

                           710       720       730       740       750       760       770       780       790       800
                    ....|....|....|....|....|....|....|....|....|....|....|....|....|....|....|....|....|....|....|....|
RferPro4_KAF7265590 DDCEKYTFCGLPLIHPKVAKKLAYSQWIPSSSPEFPQNETLKFSVLSRETLSNTKEKIVIQVSGPDKLVFYITPKTNIKVLSTSLVDILKPSKVTFQSRP
RferPro4_KAF7265591 ....................................................................................................

                           810       820       830       840       850       860       870
                    ....|....|....|....|....|....|....|....|....|....|....|....|....|....|....|...
RferPro4_KAF7265590 VYVLLHQTGKYDGTNVIFDVVVQKPPAFKGPALDIAVSGRYVHESRMHKTPAYLGFLNQFPQWTHVISWLSAYQSHLL
RferPro4_KAF7265591 ..............................................................................


                             10        20        30        40        50        60        70        80        90       100
                     ....|....|....|....|....|....|....|....|....|....|....|....|....|....|....|....|....|....|....|....|
RferPro10_KAF7278453 MNFLKIARNVRLTKSIARVASEGRKNLSHHVMEKNVKNKDLSVIVSKSRNQLYFNIKGRFLRSINGSKSFTSLRYYTTKDNGPDDLPPEDEPYNPQLPAT
RferPro10_KAF7278452 ....................................................................................................
RferPro10_KAF7278454 ....................................................................................................

                            110       120       130       140       150       160       170       180       190       200
                     ....|....|....|....|....|....|....|....|....|....|....|....|....|....|....|....|....|....|....|....|
RferPro10_KAF7278453 VAVPEVWPHVPVIAISRNIVFPRFIKLIELTNPQLIELIRRKVKLNQPYCGIFLKRNEENESEVVNNTDDVYNIGVFAQIHEMQDLGDRLRLVVMAHRRI
RferPro10_KAF7278452 ....................................................................................................
RferPro10_KAF7278454 ....................................................................................................

                            210       220       230       240       250       260       270       280       290       300
                     ....|....|....|....|....|....|....|....|....|....|....|....|....|....|....|....|....|....|....|....|
RferPro10_KAF7278453 KITGQIFDTLEDEPKE-----------------------ETDTRGRRKGRNNRLKKSFPEIKTVEEKTQTEKIMQESGESPKEPKLEPLLMAEVENVVHN
RferPro10_KAF7278452 ...............AEPKPLKLHFPAFNTTINIPKISE.............................................................
RferPro10_KAF7278454 ................P-KPLKLHFPAFNTTINIPKISE.............................................................

                            310       320       330       340       350       360       370       380       390       400
                     ....|....|....|....|....|....|....|....|....|....|....|....|....|....|....|....|....|....|....|....|
RferPro10_KAF7278453 KFRQTEEVKALTQEVIKTIRDIISLNPLYRDSLQQMMHQGQRVVDNPVYLSDLGAALTAAESKELQEVLEEMDIPKRLMLSLSLLKKEYELSKLQQKIGR
RferPro10_KAF7278452 ....................................................................................................
RferPro10_KAF7278454 ....................................................................................................

                            410       420       430       440       450       460       470       480       490       500
                     ....|....|....|....|....|....|....|....|....|....|....|....|....|....|....|....|....|....|....|....|
RferPro10_KAF7278453 EVEEKVKQHHRKYILQEQLKVIKKELGLEKEDKDAIGDKFRERIKDKVLPQAVSAVIEEELNKLNFLESHSSEFNVTRNYLDWLTSLPWGIQSEENLNLQ
RferPro10_KAF7278452 ....................................................................................................
RferPro10_KAF7278454 ....................................................................................................

                            510       520       530       540       550       560       570       580       590       600
                     ....|....|....|....|....|....|....|....|....|....|....|....|....|....|....|....|....|....|....|....|
RferPro10_KAF7278453 RASEILDQDHYGMEDIKRRILEFIAVSQLKGSTQGKILCFHGPPGVGKTSIARSIARALNREYFRFSVGGMTDVAEIKGHRRTYVGAMPGKVIQCLKKTR
RferPro10_KAF7278452 ....................................................................................................
RferPro10_KAF7278454 ....................................................................................................

                            610       620       630       640       650       660       670       680       690       700
                     ....|....|....|....|....|....|....|....|....|....|....|....|....|....|....|....|....|....|....|....|
RferPro10_KAF7278453 TENPLVLIDEVDKIGKGYSGDPSSALLELLDPEQNSNFLDHYLDVPVDLSKVLFICTANIVDTIPEPLRDRMEMIDMSGYVAEEKLAIAKQYLLPQAMKD
RferPro10_KAF7278452 ....................................................................................................
RferPro10_KAF7278454 ....................................................................................................

                            710       720       730       740       750       760       770       780       790       800
                     ....|....|....|....|....|....|....|....|....|....|....|....|....|....|....|....|....|....|....|....|
```

```
RferPro10_KAF7278453  SGLKDDKIKVEDDALNILIRNYCRESGVRNLQKHIEKVVRKVAYKVVKEETQFIDVGSQNLQEFVGKPVFTHDRMYDTTPPGVVMGLAWTAMGGSTLYVE
RferPro10_KAF7278452  ....................................................................................................
RferPro10_KAF7278454  ....................................................................................................

                             810       820       830       840       850       860       870       880       890       900
                      ....|....|....|....|....|....|....|....|....|....|....|....|....|....|....|....|....|....|....|....|
RferPro10_KAF7278453  TTTRRESSPDKDSEGSLELTGHLGDVMKESAKIALTVARNYMIKHDSSNKFLLNSHLHLHVPEGATPKDGPSAGCTIVTALLSLAKNKPIRQDLAMTGEV
RferPro10_KAF7278452  ....................................................................................................
RferPro10_KAF7278454  ....................................................................................................

                             910       920       930       940       950       960
                      ....|....|....|....|....|....|....|....|....|....|....|....|....|.
RferPro10_KAF7278453  SLMGKVLPVGGIKEKTIAAKRSGVKCIILPEENKKDFNDLPTFITEGLEVHFVSHIDQVYKIAFQE
RferPro10_KAF7278452  ..................................................................
RferPro10_KAF7278454  ..................................................................

                              10        20        30        40        50        60        70        80        90       100
                      ....|....|....|....|....|....|....|....|....|....|....|....|....|....|....|....|....|....|....|....|
RferPro16_KAF7271762  MTILVMVFLLTSLLNKSFCGFHTVKYSRSIDGGSFSTDSPCPNIFQYQTDGNGELIGLIRINPFEYSNSRIQLDVELSLGNSVHGYNGKIVLAESKDRVI
RferPro16_KAF7271763  ....................................................................................................
RferPro16_KAF7271764  ----------------------------------------------------------------------------------------------------

                             110       120       130       140       150       160       170       180       190       200
                      ....|....|....|....|....|....|....|....|....|....|....|....|....|....|....|....|....|....|....|....|
RferPro16_KAF7271762  TDIFNRKPIFYKVLFPPWQGIPPRVTKIAVNGILICNGPRIPIQIVAILTTINLQHKLSVGVTPLISSSDPTIDGSINNITPDNGD--------------
RferPro16_KAF7271763  .....................................................................................GNFYFLNWQGTSTYN
RferPro16_KAF7271764  ------------------------MV.--------------AV...........................................--------------

                             210       220       230       240       250       260       270       280       290       300
                      ....|....|....|....|....|....|....|....|....|....|....|....|....|....|....|....|....|....|....|....|
RferPro16_KAF7271762  -NSPNNPYPIDVRRDDLPTIIEIDASGDDPKYFNPFLNSNNPFNFNPQKPPPPPPPPTSTQASSPLEGKRGNPSEAFPQNPFFVGLSTPETPSKTPIQIG
RferPro16_KAF7271763  Y...................................................................................................
RferPro16_KAF7271764  -...................................................................................................

                             310       320       330       340       350       360       370       380       390       400
                      ....|....|....|....|....|....|....|....|....|....|....|....|....|....|....|....|....|....|....|....|
RferPro16_KAF7271762  NNPFFNPKTSEAPVIQNPTTVSPKVPQRGNTDIDALANVDNICGRPIATNSLIVNGHSVPKGAYPWLVAIFRQLENLSLNYICSGSLISNRHVVTAAHCV
RferPro16_KAF7271763  ....................................................................................................
RferPro16_KAF7271764  ....................................................................................................

                             410       420       430       440       450       460       470       480       490       500
                      ....|....|....|....|....|....|....|....|....|....|....|....|....|....|....|....|....|....|....|....|
RferPro16_KAF7271762  KVDSRRIKPTELLCVFGKLNIRKWVLSQGEKMLEPESVTIHPDYESGSANADIAMLTFPDPIEFNKVIRPICLWSGSNDLNLVVGKEGIVVGWGRDENGD
RferPro16_KAF7271763  ....................................................................................................
RferPro16_KAF7271764  ....................................................................................................

                             510       520       530       540       550       560       570       580       590       600
                      ....|....|....|....|....|....|....|....|....|....|....|....|....|....|....|....|....|....|....|....|
RferPro16_KAF7271762  ISTAEPRQISLPIVSQLSCVRSDKNSGGAFLHITSDNTFCAGHRNGSGPCNGDSGSGFLLRRDGVFYLRGIVSTALSDATHRSCNLKEYVVFTDASKYLD
RferPro16_KAF7271763  ....................................................................................................
RferPro16_KAF7271764  ....................................................................................................


                      ....|..
RferPro16_KAF7271762  WVLSVMR
RferPro16_KAF7271763  .......
RferPro16_KAF7271764  .......

                              10        20        30        40        50        60        70        80        90       100
                      ....|....|....|....|....|....|....|....|....|....|....|....|....|....|....|....|....|....|....|....|
RferPro33_KAF7271545  MDIKIFLLFVLFIQNIVYASIHKNLKYFQIIHADDLTHKIVKRGLQESSHPYNKIKEVHLRTHGQDFRLILSPKRSILHSKFKAYSVNGDGEETHIPIDH
RferPro33_KAF7271546  ....................................................................................................
RferPro33_KAF7271547  ----------------------------------------------------------------------------------------------------

                             110       120       130       140       150       160       170       180       190       200
                      ....|....|....|....|....|....|....|....|....|....|....|....|....|....|....|....|....|....|....|....|
RferPro33_KAF7271545  DSFMEGRVFGETSSHVNMHIEDGVMTGNIHMPDDIYHIEPSWRHIPGHDNRTMITYKQSDIKFSWDPENLPEDYGSPRMCAYVKEGVELEDNTTDHHIEK
RferPro33_KAF7271546  ....................................................................................................
RferPro33_KAF7271547  ----------------------------------------------------------------------------------------------------

                             210       220       230       240       250       260       270       280       290       300
                      ....|....|....|....|....|....|....|....|....|....|....|....|....|....|....|....|....|....|....|....|
RferPro33_KAF7271545  RQTDHIEYKLTKTRCPLLLVADYRFFQEMGGSNTKTTINYLISLIDRVHKIYNDTIWQDRQEVDGFKGMGFVIKKIVVHNEPTRIKVQEAHYNMNRENWD
RferPro33_KAF7271546  ....................................................................................................
RferPro33_KAF7271547  ---------------------------------------------------------------------------------------------.RTK---

                             310       320       330       340       350       360       370       380       390       400
                      ....|....|....|....|....|....|....|....|....|....|....|....|....|....|....|....|....|....|....|....|
RferPro33_KAF7271545  VRNLLEAFASAPQDGKYCLAHLFTHHTFRTRNSVVLGLAYIASSRINQQGGICSNEYFKNGYTLYLNSGLSSSRNHYGQRVITREADLVTAHEFGHNWGS
RferPro33_KAF7271546  ......V.SREFTHKDF.......DLK.EGG---I.....VG.P.R.SV....TP.............................................
RferPro33_KAF7271547  -----QV.SREFTHKDF.......DLK.EGG---I.....VG.P.R.SV....TP.............................................

                             410       420       430       440       450       460       470       480       490       500
                      ....|....|....|....|....|....|....|....|....|....|....|....|....|....|....|....|....|....|....|....|
RferPro33_KAF7271545  EHDPDIPECSPSASQGGSYLMYTYSVSGYDVNNKRFSPCSLRSIRKVLQAKSEKCFSEPEESFCGNLRVEGDEECDAGLLGTEDNDACCDKDCKLRPKAV
RferPro33_KAF7271546  ....................................................................................................
RferPro33_KAF7271547  ....................................................................................................

                             510       520       530       540       550       560       570       580       590       600
                      ....|....|....|....|....|....|....|....|....|....|....|....|....|....|....|....|....|....|....|....|
RferPro33_KAF7271545  CSDKNSPCCQNCQYMRSGVKCRDAQYATCEEESTCTGHSSDCPKSPPMQDGTKCQERGKCSGGKCIPYCETQGLQSCMCDIIENACKRCCKSTSNGTCSP
RferPro33_KAF7271546  ....................................................................................................
RferPro33_KAF7271547  ....................................................................................................

                             610       620       630       640       650       660       670       680       690       700
                      ....|....|....|....|....|....|....|....|....|....|....|....|....|....|....|....|....|....|....|....|
RferPro33_KAF7271545  VDSADILADGTPCIQGFCNNGNCEKTVQDVVERFWDIIEDININKVLLFLRDNIVGTVVLITAFLWLPASCIIGYVDRKRRKEELKRWEWRNNSQELIHN
```

```
RferPro33_KAF7271546 ....................................................................................................
RferPro33_KAF7271547 ....................................................................................................

                       710       720       730
                   ....|....|....|....|....|....|.
RferPro33_KAF7271545 SEKVKNIIKIRVTNKENRNPQSEQSPTKHSE
RferPro33_KAF7271546 ...............................
RferPro33_KAF7271547 ...............................

                        10        20        30        40        50        60        70        80        90       100
                   ....|....|....|....|....|....|....|....|....|....|....|....|....|....|....|....|....|....|....|....|
RferPro38_KAF7280661 MSSVRCSLATAGKYAFATRSSKIQQGILSAIYQKQINQNKHISTSVILLKTFGAGEPPTNTTPPPNSSTNSGGNGKNKNTLSCPKCGDPCTHVETFVSST
RferPro38_KAF7280662 ....................................................................................................

                       110       120       130       140       150       160       170       180       190       200
                   ....|....|....|....|....|....|....|....|....|....|....|....|....|....|....|....|....|....|....|....|
RferPro38_KAF7280661 RFVKCEKCHHFFVVLSEVDSKKKEGVDLKNGQYRKPPPPPKKIYDYLNKHVVGQEYAKKVLSVAVYNHYKRIYNNTPNQVNNRQDMAVMEQGPHHNITHR
RferPro38_KAF7280662 ....................................................................................................

                       210       220       230       240       250       260       270       280       290       300
                   ....|....|....|....|....|....|....|....|....|....|....|....|....|....|....|....|....|....|....|....|
RferPro38_KAF7280661 ------GMGHNSMGLSFSPTEPPYSKTTSQSAVGSDILDRTTHELKLEKSNILLLGPTGSGKTLLAQTIAQCLDVPFAICDCTTLTQAGYVGEDIESVIG
RferPro38_KAF7280662 DLLHIT..........................................................................................

                       310       320       330       340       350       360       370       380       390       400
                   ....|....|....|....|....|....|....|....|....|....|....|....|....|....|....|....|....|....|....|....|
RferPro38_KAF7280661 KLLQDAGYSVEKAQIGIVFLDEVDKIGAVPGIHQLRDVGGEGVQQGMLKMLEGTVVNVPERNSPRKLRGETIQVDTTNILFVASGAYNGLERLIQRRNNE
RferPro38_KAF7280662 ....................................................................................................

                       410       420       430       440       450       460       470       480       490       500
                   ....|....|....|....|....|....|....|....|....|....|....|....|....|....|....|....|....|....|....|....|
RferPro38_KAF7280661 NYLGFGAPVSTGQGRRAVAQEATIHSSTQSAEEENTEKDAALRQVQARDLIDFGMIPEFVGRFPVLVPFHSLDHVMLRLLAMDQCQLTFSPDALKAIAAL
RferPro38_KAF7280662 ....................................................................................................

                       510       520       530       540       550       560       570
                   ....|....|....|....|....|....|....|....|....|....|....|....|....|....|
RferPro38_KAF7280661 AMERKTGARGLRAIMETLLLEPMFEVPGAGINEVHITEDCVRGKCPPIYVKTNPEGSEEDEINATIRLKQ
RferPro38_KAF7280662 ......................................................................

                        10        20        30        40        50        60        70        80        90       100
                   ....|....|....|....|....|....|....|....|....|....|....|....|....|....|....|....|....|....|....|....|
RferPro42_KAF7280186 MVTSFNSDNAMEAQVVSRERSFFHSRLNSPSSSSECNVQDVNTSDVEGKVKNRFLSIWNNMKYELNSVGMKLKPNFSKESPVWLLGMCYRKIEPPNPNST
RferPro42_KAF7280185 ...............................................................----.................................
RferPro42_KAF7280187 ...............................................................-----................................

                       110       120       130       140       150       160       170       180       190       200
                   ....|....|....|....|....|....|....|....|....|....|....|....|....|....|....|....|....|....|....|....|
RferPro42_KAF7280186 ELGTDVAAFQSQSELANNEDEGLEGFRKDFISKLWLTYRREFPILNGSTYSSDCGWGCMIRSGQMLIAQALVIHFLGRDWRWNPDKRELNYHQKIIKWFG
RferPro42_KAF7280185 ....................................................................................................
RferPro42_KAF7280187 ....................................................................................................

                       210       220       230       240       250       260       270       280       290       300
                   ....|....|....|....|....|....|....|....|....|....|....|....|....|....|....|....|....|....|....|....|
RferPro42_KAF7280186 DKPSINSPLSLHSLVRIGEGLGKKAGDWYGPGLVAHLFKKAFEDAAKENSEFDHLTVCVAQNCTVYIRDVFVECMGKSNNWKSLILLIPVRLGTEKFNTI
RferPro42_KAF7280185 ....................................................................................................
RferPro42_KAF7280187 ....................................................................................................

                       310       320       330       340       350       360       370       380       390       400
                   ....|....|....|....|....|....|....|....|....|....|....|....|....|....|....|....|....|....|....|....|
RferPro42_KAF7280186 YAPCLTTLLSIKECIGIIGGRPKHSLYFVGYQDDKLIHLDPHYCQEVVDVWAEDFPLTTFHCRSPRKLPINKIDPSCCIGFYCATKQDFLNLIETVQPIV
RferPro42_KAF7280185 ....................................................................................................
RferPro42_KAF7280187 ....................................................................................................

                       410       420       430       440       450
                   ....|....|....|....|....|....|....|....|....|....|....
RferPro42_KAF7280186 VPPSGANDYPMFTFCDGFSCDAESDVKDFLPSECEYQNNTSDLEDIESEAFELV
RferPro42_KAF7280185 ......................................................
RferPro42_KAF7280187 ......................................................

                        10        20        30        40        50        60        70        80        90       100
                   ....|....|....|....|....|....|....|....|....|....|....|....|....|....|....|....|....|....|....|....|
RferPro47_KAF7265675 MKLLWVLTILFAITVLPKGTLAVANGTVDEPHSRPWIAMVSASISLLEYQYCAGILISLNYILTVASCFHTDSKYVQIHSLGTYLGVYNWDTLEGFVQMS
RferPro47_KAF7265674 ........L....A.F.N......................LPT...V.........................D..IK......F......Y.......A.

                       110       120       130       140       150       160       170       180       190       200
                   ....|....|....|....|....|....|....|....|....|....|....|....|....|....|....|....|....|....|....|....|
RferPro47_KAF7265675 GTIPINKVYQHENFNATTFENDIAIIELDIPARPIKGLVEIANLATEEDVQLGQVGRVSGWYYPIRESNQGLSHGGEVTVVDNSVCQETYPSYAISENVI
RferPro47_KAF7265674 ........H....................................................H..V..............................N....

                       210       220       230       240       250       260
                   ....|....|....|....|....|....|....|....|....|....|....|....|....
RferPro47_KAF7265675 CASGYVAFCNGDQGTGLLINDKVVGMGVGPKSVLVPTEQCPIIPNTYLYIPKYLDWIKAHSDLE
RferPro47_KAF7265674 ....................G.I....I.......SA....VV.....................

                        10        20        30        40        50        60        70        80        90       100
                   ....|....|....|....|....|....|....|....|....|....|....|....|....|....|....|....|....|....|....|....|
RferPro56_KAF7287182 MADKQLGMAALVVVFFSLIVVVAPATNNAMLYLSQYGYFGPLKANSSQLVDENTYKKAIEEFQSFAGIPVTGELDKETLQTMEMPRCGVKDKVGTGANRA
RferPro56_KAF7287184 ....................................................................................................
RferPro56_KAF7287183 ....................................................................................................

                       110       120       130       140       150       160       170       180       190       200
                   ....|....|....|....|....|....|....|....|....|....|....|....|....|....|....|....|....|....|....|....|
RferPro56_KAF7287182 KRYALQGSRWKVKNLSYKISKYPPKLKRAEVDAEIQRAFNVWSEFTDLTFTPKTGQVHIEIRFESGEHGDGDPFDGPGGTLAHAYFP-------------
RferPro56_KAF7287184 .......................................................................................-------------
RferPro56_KAF7287183 .......................................................................................PPESQYLTSSQHM

                       210       220       230       240       250       260       270       280       290       300
```

```
                          ....|....|....|....|....|....|....|....|....|....|....|....|....|....|....|....|....|....|....|....|
RferPro56_KAF7287182     ----------------------------------VFGGDAHFDATESWSINSYRGTNLFQVAAHEFGHSLGLSHSDVREALMAPFYRGYNPIFNLDQDDI
RferPro56_KAF7287184     ----------------------------------..................................................................
RferPro56_KAF7287183     YKLGVGTNHQSLPWYNPYFVGWYHPNPPTYNPGK..................................................................

                                310       320       330       340       350       360       370       380       390       400
                          ....|....|....|....|....|....|....|....|....|....|....|....|....|....|....|....|....|....|....|....|
RferPro56_KAF7287182     DGIQALYGRKTSKSKQPDDTEPTDESKYTPKSIPGSSSGHGLCEDPKFDTIFNSAEGYTFIFKGDQYYKLTEESVATGYPKLISEGWPGLPGNIDAAFTY
RferPro56_KAF7287184     ....................................................................................................
RferPro56_KAF7287183     ....................................................................................................

                                410       420       430       440       450       460       470       480       490       500
                          ....|....|....|....|....|....|....|....|....|....|....|....|....|....|....|....|....|....|....|....|
RferPro56_KAF7287182     KNGKTYFFKGSKYWRYKGRKIDGDYPKDISEGFSGIPNNIDTAMVWSGNGKIYFFKGSKFYRFDPTQKPPVPKTFPKPISNWEGIPDNLDAAFQWTNGYT
RferPro56_KAF7287184     ....................................................................................................
RferPro56_KAF7287183     ....................................................................................................

                                510       520       530       540       550       560       570       580       590       600
                          ....|....|....|....|....|....|....|....|....|....|....|....|....|....|....|....|....|....|....|....|
RferPro56_KAF7287182     YFYKAGAYYRFNDRAFAVDATTPAFPRSTAYWWFGCSNAPSGTVATGESRGWLVGEESGPQDNNGFDIDAGDHQDPPTNDTPLHQPDGDDGNNGSSVLDG
RferPro56_KAF7287184     ......................................................................VVNRIFRS------------MF.RAGYIPE
RferPro56_KAF7287183     ......................................................................-----------------------------E

                                610       620       630       640       650       660       670       680
                          ....|....|....|....|....|....|....|....|....|....|....|....|....|....|....|....|....|.
RferPro56_KAF7287182     EAKSIERQNEAASGARLRPLEGARVGGARGDRAALAAARAAAADALSQDGGRAEEPAAAPGAAAVQKCRVMRESAAAASPSEHAAP
RferPro56_KAF7287184     NYRK.P.RQ-----------------------------------------------------------------------------
RferPro56_KAF7287183     NYRK.P.RQ-----------------------------------------------------------------------------

                                  10        20        30        40        50        60        70        80        90       100
                            ....|....|....|....|....|....|....|....|....|....|....|....|....|....|....|....|....|....|....|....|
RferPro70a_KAF7274333      MDTIQSISSAIQDKMTRYTNADFGDDDSVNSVQLTEGISTSATHIRYHPGARLWQVRSTLERILLFLGTFLFMVIIVLSIVINVVEHHLQMSKLEQARFV
RferPro70a_KAF7274332      .EFFRRLVG--R.T......................................................................................

                                 110       120       130       140       150       160       170       180       190       200
                            ....|....|....|....|....|....|....|....|....|....|....|....|....|....|....|....|....|....|....|....|
RferPro70a_KAF7274333      AEQPCLNESCVIISSHMLEAMDLSVDPCDDFYAYSCNGWVKANPIPDGKSNWGTFMKLEQQNWLVIKTVLEQPIDTFKSKAEQKAKMYYESCLDTNDTVE
RferPro70a_KAF7274332      ....................................................................................................

                                 210       220       230       240       250       260       270       280       290       300
                            ....|....|....|....|....|....|....|....|....|....|....|....|....|....|....|....|....|....|....|....|
RferPro70a_KAF7274333      TLGAEPMFDLLRKLGGWNITDAPFNISKWTLQNTLEIVQNKYGVGALFSYGVGEDDRNSSRHVLQIDQSGLALPTRDNYLNKTADHIKVLNAYLEYMTKV
RferPro70a_KAF7274332      ....................................................................................................

                                 310       320       330       340       350       360       370       380       390       400
                            ....|....|....|....|....|....|....|....|....|....|....|....|....|....|....|....|....|....|....|....|
RferPro70a_KAF7274333      GVLLGGDLNATKKQMEDVIQFETKLANITTPSDLRRDEESLYHLMTVAELQEKANFIDWRQFFENAMKVVQKKISSKQQIVVYAPDYLGNLTNLINDYQN
RferPro70a_KAF7274332      ....................................................................................................

                                 410       420       430       440       450       460       470       480       490       500
                            ....|....|....|....|....|....|....|....|....|....|....|....|....|....|....|....|....|....|....|....|
RferPro70a_KAF7274333      TTEGRIILNNYLVWQTVKVFTLCLSKPFRDAYKGLRTALMGSDGADEPQWRYCIQDTNNVLGFALGAIFVREVFHSDSKEQAEVMINNVRDAFKRNFQNL
RferPro70a_KAF7274332      ....................................................................................................

                                 510       520       530       540       550       560       570       580       590       600
                            ....|....|....|....|....|....|....|....|....|....|....|....|....|....|....|....|....|....|....|....|
RferPro70a_KAF7274333      NWMDEDTRKVAIDKADAISDMIGYPEFIKNVNHLDERFDNLTVRSDRYFENNVNINYYNLKKNLEKINEPVNKTTWSMVPSTVNAYYTPTKNQMVFPAGI
RferPro70a_KAF7274332      ....................................................................................................

                                 610       620       630       640       650       660       670       680       690       700
                            ....|....|....|....|....|....|....|....|....|....|....|....|....|....|....|....|....|....|....|....|
RferPro70a_KAF7274333      LQNPFYDPRFPDSLNYGAMGVVMGHELTHGFDDQGRQYDKNGNLNHWWNNKTVERFKERTKCVVQQYNNYKINGKNVNGNQTLGENIADNGGLKAAYHAY
RferPro70a_KAF7274332      ....................................................................................................

                                 710       720       730       740       750       760       770       780
                            ....|....|....|....|....|....|....|....|....|....|....|....|....|....|....|....|....|...
RferPro70a_KAF7274333      MDMMKNKQEPRKLPGLPLNHRQLFFVAFAQVWCSAFTKEATNLEIEKDTHSPAKFRVIGALSNLREFSDEFQCKVGTRMNPEKKCEVW
RferPro70a_KAF7274332      ........................................................................................

                                  10        20        30        40        50        60        70        80        90       100
                            ....|....|....|....|....|....|....|....|....|....|....|....|....|....|....|....|....|....|....|....|
RferPro70b_KAF7279498      -------MTSSVKQTVIK-------NPTWWKRRTSMERCLTLITIVVFIIGISLIVALASVLYNRNKEVANSQFTAEALHGQKTFVITAPDEESSNPKLC
RferPro70b_KAF7279497      MAPMNFANV.Y.GVPNK.NAPPSDT...........................................................................

                                 110       120       130       140       150       160       170       180       190       200
                            ....|....|....|....|....|....|....|....|....|....|....|....|....|....|....|....|....|....|....|....|
RferPro70b_KAF7279498      LSAGCIHTASKVLEYMDQSVDPCDDFYQFTCGNFLKKTNIPDDKSSVTSFSIINDNLQEQLRSMIEEPIQPNEPKPFQLTKKLYRACMNKTLIEEEGLTN
RferPro70b_KAF7279497      ....................................................................................................

                                 210       220       230       240       250       260       270       280       290       300
                            ....|....|....|....|....|....|....|....|....|....|....|....|....|....|....|....|....|....|....|....|
RferPro70b_KAF7279498      INRILKQLGGWPVIDGDQWFDGDFDWRESVYKFRKLGYSVDYFIDFSVGIDVKNSTKRVIDLDQASLGLRREFLTKGLEDKLVKAYYDYMVDIAVLFGAD
RferPro70b_KAF7279497      ....................................................................................................

                                 310       320       330       340       350       360       370       380       390       400
                            ....|....|....|....|....|....|....|....|....|....|....|....|....|....|....|....|....|....|....|....|
RferPro70b_KAF7279498      KGRATKELRESLDFEIQLANISLPSEKRRNATALYNPMTVEELEFKFPSIPWMEYINTLLAPDIQISNQEMIVVSVPKYITDFEALISRTPKRVQANYVM
RferPro70b_KAF7279497      ....................................................................................................

                                 410       420       430       440       450       460       470       480       490       500
                            ....|....|....|....|....|....|....|....|....|....|....|....|....|....|....|....|....|....|....|....|
RferPro70b_KAF7279498      WRAAASSVSYLNDNLRKRQLEYTTIVTGRTEREARWKECIDISAGSLSIASGALYVRKYFNEEARQNAKEMVADIRAEFETILKSVDWMDDETRKNALDK
RferPro70b_KAF7279497      ....................................................................................................

                                 510       520       530       540       550       560       570       580       590       600
                            ....|....|....|....|....|....|....|....|....|....|....|....|....|....|....|....|....|....|....|....|
RferPro70b_KAF7279498      AKSMSTHIAYPDELLDNKKLEEFYDALELDENQYLNSILNLTLFGTRLSFKRLRQPVNKTDWITHGRPAVVNAFYSAIENSIQFPAGILQGVFFSADRPR
```

```
RferPro70b_KAF7279497  ....................................................................................................

                            610       620       630       640       650       660       670       680       690       700
                       ....|....|....|....|....|....|....|....|....|....|....|....|....|....|....|....|....|....|....|....|
RferPro70b_KAF7279498  YMNYGAIGFVIGHEITHGFDDQGRQFDKEGNLVDWWQSGTKKAFVEKAQCIIDQYGNYTVPELKLHLNGINTQGENIADNGGIKQAYLAYRKWISRNGEE
RferPro70b_KAF7279497  ....................................................................................................

                            710       720       730       740       750       760       770       780       790       800
                       ....|....|....|....|....|....|....|....|....|....|....|....|....|....|....|....|....|....|....|
RferPro70b_KAF7279498  PSLPGLKYSARQMFWISAASIWCSETREEELRQLVVTDEHAPDRYRVIVPLSNMKYFADDFKCPVGSKMNPVKKCQLNGINTQGENIADNGGIKEAYLAY
RferPro70b_KAF7279497  ....................................................................................................

                            810       820       830       840       850       860       870       880
                       ....|....|....|....|....|....|....|....|....|....|....|....|....|....|....|....|....|...
RferPro70b_KAF7279498  QNWIRRNGKEPQLPGLKYTPNQMFWISASNTWCSKYRPESLKLRVLTGYHSPGQFRVQGPFSNSEFFARDFQCPVGSQMNPERKCQVW
RferPro70b_KAF7279497  ........................................................................................

                             10        20        30        40        50        60        70        80        90       100
                       ....|....|....|....|....|....|....|....|....|....|....|....|....|....|....|....|....|....|....|
RferPro80_KAF7288074   MDNLDILRNTLLPTCLGIIPIIINYVLFTQQFYSTASELSKYAINLILDVINQQIFETFLNCLIIHRVFVILSSACSNTMKNLVFIFLAVVFYVNARVTY
RferPro80_KAF7288075   ....................................................................................................

                            110       120       130       140       150       160       170       180       190       200
                       ....|....|....|....|....|....|....|....|....|....|....|....|....|....|....|....|....|....|....|
RferPro80_KAF7288074   PNVYKPIQTLELTSDEDVGDPLILTPLLKENKIEEARQKSTVNITGFETVESYSGFFTVDENFDSNLFFWFFPSENDFQNDPVLLWLQGGPGASSLYALF
RferPro80_KAF7288075   ....................................................................................................

                            210       220       230       240       250       260       270       280       290       300
                       ....|....|....|....|....|....|....|....|....|....|....|....|....|....|....|....|....|....|....|
RferPro80_KAF7288074   CENGPLIVLNGDQLSLREYSWTKNHSVLYIDNPAGTGFSFTNGGYAQNETKVGEDLYQALVQFFTLFPEIAENPFFVTGESYAGKYIPAIGYAIYLHRNT
RferPro80_KAF7288075   ....................................................................................................

                            310       320       330       340       350       360       370       380       390       400
                       ....|....|....|....|....|....|....|....|....|....|....|....|....|....|....|....|....|....|....|
RferPro80_KAF7288074   EDIQINLKGLLIGDGLTDPENQISEYGNYLYELGFIDQNGKEEFIKYQNETIQYIQQNDFLKAFDTFDILLNGDFTSPTVFQNLTGFSSYFNYLYRPSPD
RferPro80_KAF7288075   ....................................................................................................

                            410       420       430       440       450       460       470       480       490       500
                       ....|....|....|....|....|....|....|....|....|....|....|....|....|....|....|....|....|....|....|
RferPro80_KAF7288074   DDNSYEAFLQSADVRKAIHVGNLSYSSENVEENLREDLLQSVAPWVAELLSNYRMLFFNGQLDIIVAYPLTINFLNNLEFSGSDEYKTAPRSIWTVDDEV
RferPro80_KAF7288075   ....................................................................................................

                            510       520       530       540       550       560       570       580       590       600
                       ....|....|....|....|....|....|....|....|....|....|....|....|....|....|....|....|....|....|....|
RferPro80_KAF7288074   AGYVKVAANLTEVLVRNSGHMVPMDQPKWAYDLVYKFFGSEKVQSRGEKRAVHKVQYIRKRTVNKRWWRNTKNKLKSITTENTVKSSTEKMFRFIFIAIL
RferPro80_KAF7288075   .....................................LYFKRIN-------------------------------------K..................

                            610       620       630       640       650       660       670       680       690       700
                       ....|....|....|....|....|....|....|....|....|....|....|....|....|....|....|....|....|....|....|
RferPro80_KAF7288074   LVTLILDTESRSFISKVYPKFKQSQPEIGEFPGVPVILTPLIESGKIYDARLASQVQFNGFLGKTSFSGYFTVDKTYESNLFFWYFPCETGDKDAPLLLW
RferPro80_KAF7288075   ....................................................................................................

                            710       720       730       740       750       760       770       780       790       800
                       ....|....|....|....|....|....|....|....|....|....|....|....|....|....|....|....|....|....|....|
RferPro80_KAF7288074   LQGGPGATSLIGIFVENGPFETKFEHGLRERTTSWTKNHSVIYIDNPVGTGYSFTTGGYAQNQTIVGEHIYRALKQFFTVFPEEKKKDFYITGESYAGKY
RferPro80_KAF7288075   ....................................................................................................

                            810       820       830       840       850       860       870       880       890       900
                       ....|....|....|....|....|....|....|....|....|....|....|....|....|....|....|....|....|....|....|
RferPro80_KAF7288074   IPAVAYTIYQKNSGQNETINLKGLAIGDGFCDPINQLEYADYLYQIGLYDASTRDNIKNLEKQGLQMIQNKQFAEAARFFDSLMDGDFSNTTKFKNATGF
RferPro80_KAF7288075   ....................................................................................................

                            910       920       930       940       950       960       970       980       990      1000
                       ....|....|....|....|....|....|....|....|....|....|....|....|....|....|....|....|....|....|....|
RferPro80_KAF7288074   DNYDNFLYPIDPLNTGMELMAKYIVQDDVRAAIHVGNATFNEDSAVEENLLEDLMQSVTPWITELLKHYKILFYNGQLDIIVGYPLTVNFLNHLDFNAAT
RferPro80_KAF7288075   ....................................................................................................

                           1010      1020      1030      1040      1050      1060      1070      1080      1090      1100
                       ....|....|....|....|....|....|....|....|....|....|....|....|....|....|....|....|....|....|....|
RferPro80_KAF7288074   EYQTAARHQWIVDSNIAGYVKQAGNLTEILVRNAGHMVPADQPEWALDMITRFSHGSLVYITTNSKMNTVFFIFGLYIIKQSEGYYKDKPLPHRELEDVG
RferPro80_KAF7288075   ....................................................................................................

                           1110      1120      1130      1140      1150      1160      1170      1180      1190      1200
                       ....|....|....|....|....|....|....|....|....|....|....|....|....|....|....|....|....|....|....|
RferPro80_KAF7288074   EPLIVTPYIENGSINEAQQKAKVTYSELTKNNLTSYSGYFTVDKTYNSNLFFWFFLSENNPSEDPVILWLQGGPGASSLYGLFTENGPYEVNENLELSYR
RferPro80_KAF7288075   ....................................................................................................

                           1210      1220      1230      1240      1250      1260      1270      1280      1290      1300
                       ....|....|....|....|....|....|....|....|....|....|....|....|....|....|....|....|....|....|....|
RferPro80_KAF7288074   DYSWTRDHSVLFIDNPAGTGFSFTDSGFAQNETKVGDDLYSALVQFFTLFPDLQNNDFYVTGESYGGKYVPSISYTIHKNNPGADLKINLQGFAIGNPLS
RferPro80_KAF7288075   ....................................................................................................

                           1310      1320      1330      1340      1350      1360      1370      1380      1390      1400
                       ....|....|....|....|....|....|....|....|....|....|....|....|....|....|....|....|....|....|....|
RferPro80_KAF7288074   DPINQADYGSYMYYLGLIDSNLKTNLKSLHAMFTCAIRRQEYEKATNLLETIRSKMHNSNGLPNVYNHQAIEEEDPEYFATYLSQDELRPQVHVGNVEYG
RferPro80_KAF7288075   ....................................................................................................

                           1410      1420      1430      1440      1450      1460      1470      1480      1490      1500
                       ....|....|....|....|....|....|....|....|....|....|....|....|....|....|....|....|....|....|....|
RferPro80_KAF7288074   ISSDTVYDNLLADKSKSIAPWIEELLNNYRALFYNGQLDTADPYPLMVNFLKKLSFNGHDEYLAGERQIWRVGDEIAGYWKTGGYLTEVLVRDAGHMVPT
RferPro80_KAF7288075   ....................................................................................................

                           1510      1520
                       ....|....|....|....|
RferPro80_KAF7288074   FQPKWSYDLIYKFVRNTTLD
RferPro80_KAF7288075   ....................
                              10        20        30        40        50        60        70        80        90       100
```

```
                     ....|....|....|....|....|....|....|....|....|....|....|....|....|....|....|....|....|....|....|....|
RferPro105_KAF7266901  MDSERKCESSGCGKSAKLQCPTCIKLGIPGSFFCSQECFKSNWKSHKIIHSLA-----KGEGNNKKDDTFNPWPYYNFTGRLRPYPQSSKRTVPLEIGRP
RferPro105_KAF7266902  .....................................................SNNLT..........................................

                             110       120       130       140       150       160       170       180       190       200
                     ....|....|....|....|....|....|....|....|....|....|....|....|....|....|....|....|....|....|....|
RferPro105_KAF7266901  DYADHPQGYPLSEQAVKGSGHIKVLDDEEIEGMKVACKLGREVLDEAARVCDVGVTTDEIDRIVHEACIERDCYPSPLNYYEFPASCCTSVNEVICHGIP
RferPro105_KAF7266902  ....................................................................................................

                             210       220       230       240       250       260       270       280       290       300
                     ....|....|....|....|....|....|....|....|....|....|....|....|....|....|....|....|....|....|....|
RferPro105_KAF7266901  DLRPLQDGDICNVDVTVYHRGFHGDLNETFFVGNVNDKVKNLVKVTHECLMKAIEIVKPGEKYREIGNVIQKHAQTHGFSVVRSYCGHGIHRLFHTAPNV
RferPro105_KAF7266902  ....................................................................................................

                             310       320       330       340       350       360       370       380
                     ....|....|....|....|....|....|....|....|....|....|....|....|....|....|....|....|....
RferPro105_KAF7266901  PHYAKNRAVGVIKPGHCFTIEPMISVGTFKDEMWPDKWTAVTADGQWSAQFEQTLLVNETGCEILTIRREKNGQPHFMDKITHH
RferPro105_KAF7266902  ....................................................................................

                              10        20        30        40        50        60        70        80        90       100
                     ....|....|....|....|....|....|....|....|....|....|....|....|....|....|....|....|....|....|....|
RferPro115_KAF7281067  MRFFILFILAVEGIYGRQGFGPTDQEWGFAQVREGAHIFWWLHYTTATTEPTERPLIIWLQGGPGGSSTSYGNFAELGPLDLDLNPRNTSWVHHANVLFV
RferPro115_KAF7281068  ....................................................................................................

                             110       120       130       140       150       160       170       180       190       200
                     ....|....|....|....|....|....|....|....|....|....|....|....|....|....|....|....|....|....|....|
RferPro115_KAF7281067  DNPVGTGFSYVEQNSQFTTTNAQIADDFVAFLQQFYAELPSFKQVPLYIFCESYGGKMTAQIALHLNQAIEAGKLDINFKGIGLGDSWISPVDSCITWAP
RferPro115_KAF7281068  ....................................................................................................

                             210       220       230       240       250       260       270       280       290       300
                     ....|....|....|....|....|....|....|....|....|....|....|....|....|....|....|....|....|....|....|
RferPro115_KAF7281067  YLYQLGVIDTQQYVKLDSQAQTLKALVDDGEWLEATQAWEALESKVTSYGFCIDFYNILTKCEDLYFRVRKPGVSDALKSYDEDTIMNEMVRPALNISQY
RferPro115_KAF7281068  .........................................................................--.........................

                             310       320       330       340       350       360       370       380       390       400
                     ....|....|....|....|....|....|....|....|....|....|....|....|....|....|....|....|....|....|....|
RferPro115_KAF7281067  WGTQSSEVFHYLETDFMKPVTDVVEQLLNETSIKVAVYNGQLDLIVDTVGTVNWVDRLRFKEAEEWQKNTRTIFLINNYYEGYLKKAGNLVFYWVLRAGH
RferPro115_KAF7281068  ....................................................................................................

                             410       420
                     ....|....|....|....|...
RferPro115_KAF7281067  MVPSDNPNGMLYILQQVTDNFSV
RferPro115_KAF7281068  .......................

                              10        20        30        40        50        60        70        80        90       100
                     ....|....|....|....|....|....|....|....|....|....|....|....|....|....|....|....|....|....|....|
RferPro117_KAF7283114  MLFQISVGDSFSWLPDRKMNLFLVLVWCLCLVTGKSGFGGTDEKWGYAKVNEKAHMFWWLYYTTATSDYTQRPLVIWLQGGPGGSSTGIGNFLEIGPVDV
RferPro117_KAF7283115  ....................................................................................................

                             110       120       130       140       150       160       170       180       190       200
                     ....|....|....|....|....|....|....|....|....|....|....|....|....|....|....|....|....|....|....|
RferPro117_KAF7283114  TQQPREINWISYLNVLFVDNPVGTGFSYVENNDTAYFAKNNTAIAQNFLVFLQGFLNENSEFQKVPIYIFGQSYGGKMAVDIAWLINQESKAGNVNCNLK
RferPro117_KAF7283115  .........................--.........................................................................

                             210       220       230       240       250       260       270       280       290       300
                     ....|....|....|....|....|....|....|....|....|....|....|....|....|....|....|....|....|....|....|
RferPro117_KAF7283114  GIALGDSWISPIDSLISWPNYLYNLGFLDTEEFNTLKTVTDYIQEDVANGNFENATQYFHATQFIINKFTSVDYYNVMKEQNKVYNLRNSEWKENPEDVY
RferPro117_KAF7283115  ....................................................................................................

                             310       320       330       340       350       360       370       380       390       400
                     ....|....|....|....|....|....|....|....|....|....|....|....|....|....|....|....|....|....|....|
RferPro117_KAF7283114  VLKGIIPDDIFESLINIKQVLKSQDEDDHQTLYKLMNGPVKTALNISDILPIEWGEQSDMVFRAVDLDFMKPVTELVERLLNETDIEVSVYNGQLDLIVD
RferPro117_KAF7283115  ....................................................................................................

                             410       420       430       440       450       460       470
                     ....|....|....|....|....|....|....|....|....|....|....|....|....|....|....
RferPro117_KAF7283114  TPGTVAWVNKLHWRYSETWKETERIPIVIDNIYEGYQKRYKNFNLYWVHRAGHSVPADNPHTMASILKQITHID
RferPro117_KAF7283115  ..........................................................................

                              10        20        30        40        50        60        70        80        90       100
                     ....|....|....|....|....|....|....|....|....|....|....|....|....|....|....|....|....|....|....|
RferPro119_KAF7286333  MSNMRYVLAFAVFSCCIFRSN-FLPTSGERKSSSDVLDFMKKFGYVVDEGGEAEALYTEEGLKSSIMTMQKFGGIEQTGIIDNATLKLITSPRCGVPDII
RferPro119_KAF7286332  -----M..KEL.IL..LTTCYTC..VP.KPVPITKA................................................................

                             110       120       130       140       150       160       170       180       190       200
                     ....|....|....|....|....|....|....|....|....|....|....|....|....|....|....|....|....|....|....|
RferPro119_KAF7286333  ANNRKKRFALIGGWNKRNITYFISNYSPKLGEEVVANNIQKALDLWGSYGRLTFTRVYNQYADIIVAFATGDHGDGNPFDGPGLILAHAFFPQNSESTGI
RferPro119_KAF7286332  ....................................................................................................

                             210       220       230       240       250       260       270       280       290       300
                     ....|....|....|....|....|....|....|....|....|....|....|....|....|....|....|....|....|....|....|
RferPro119_KAF7286333  GGDIHFDNDEDWADIPNHVGNKEGTDFFSVALHELGHSLGLSHSSIESSIMFPYYTAYDPDKPIALDYDDIMGMYHLYISKTVPDDKYNQNSPTEAYYPT
RferPro119_KAF7286332  ....................................................................................................

                             310       320       330       340       350       360       370       380       390       400
                     ....|....|....|....|....|....|....|....|....|....|....|....|....|....|....|....|....|....|....|
RferPro119_KAF7286333  TGTESTTRSDHPTYWPTTTNRVTVSYDGDVETVDVHKEHEWQHLTPRTKPSSIGHICNGHFDAVATLRGELFIFKDKYIWRLRERGQILRGYPTKIRDMF
RferPro119_KAF7286332  ....................................................................................................

                             410       420       430       440       450       460       470       480       490       500
                     ....|....|....|....|....|....|....|....|....|....|....|....|....|....|....|....|....|....|....|
RferPro119_KAF7286333  PFLPKDVNKIDAAYERGDGNIIFFAGKQFWVSDGTRLLENSPRPLTDYGLPDNLENIDAVQLWGLNHKVYIYKNDRFWRYNETSKTMDQGYPMHMDRWPG
RferPro119_KAF7286332  ....................................................................................................

                             510       520       530       540       550       560
                     ....|....|....|....|....|....|....|....|....|....|....|....|...
```

```
RferPro119_KAF7286333  VPHNLDAATTWIDGITYFFKDELFWKFDNEWIRASGSSPLPVGPLWLGCKEDPDEIVRLFGSD
RferPro119_KAF7286332  ...............................................................

                                10        20        30        40        50        60        70        80        90       100
                       ....|....|....|....|....|....|....|....|....|....|....|....|....|....|....|....|....|....|....|....|
RferPro120_KAF7288074  MDNLDILRNTLLPTCLGIIPIIINYVLFTQQFYSTASELSKYAINLILDVINQQIFETFLNCLIIHRVFVILSSACSNTMKNLVFIFLAVVFYVNARVTY
RferPro120_KAF7288075  ....................................................................................................

                               110       120       130       140       150       160       170       180       190       200
                       ....|....|....|....|....|....|....|....|....|....|....|....|....|....|....|....|....|....|....|....|
RferPro120_KAF7288074  PNVYKPIQTLELTSDEDVGDPLILTPLLKENKIEEARQKSTVNITGFETVESYSGFFTVDENFDSNLFFWFFPSENDFQNDPVLLWLQGGPGASSLYALF
RferPro120_KAF7288075  ....................................................................................................

                               210       220       230       240       250       260       270       280       290       300
                       ....|....|....|....|....|....|....|....|....|....|....|....|....|....|....|....|....|....|....|....|
RferPro120_KAF7288074  CENGPLIVLNGDQLSLREYSWTKNHSVLYIDNPAGTGFSFTNGGYAQNETKVGEDLYQALVQFFTLFPEIAENPFFVTGESYAGKYIPAIGYAIYLHRNT
RferPro120_KAF7288075  ....................................................................................................

                               310       320       330       340       350       360       370       380       390       400
                       ....|....|....|....|....|....|....|....|....|....|....|....|....|....|....|....|....|....|....|....|
RferPro120_KAF7288074  EDIQINLKGLLIGDGLTDPENQISEYGNYLYELGFIDQNGKEEFIKYQNETIQYIQQNDFLKAFDTFDILLNGDFTSPTVFQNLTGFSSYFNYLYRPSPD
RferPro120_KAF7288075  ....................................................................................................

                               410       420       430       440       450       460       470       480       490       500
                       ....|....|....|....|....|....|....|....|....|....|....|....|....|....|....|....|....|....|....|....|
RferPro120_KAF7288074  DDNSYEAFLQSADVRKAIHVGNLSYSSENVEENLREDLLQSVAPWVAELLSNYRMLFFNGQLDIIVAYPLTINFLNNLEFSGSDEYKTAPRSIWTVDDEV
RferPro120_KAF7288075  ....................................................................................................

                               510       520       530       540       550       560       570       580       590       600
                       ....|....|....|....|....|....|....|....|....|....|....|....|....|....|....|....|....|....|....|....|
RferPro120_KAF7288074  AGYVKVAANLTEVLVRNSGHMVPMDQPKWAYDLVYKFFGSEKVQSRGEKRAVHKVQYIRKRTVNKRWWRNTKNKLKSITTENTVKSSTEKMFRFIFIAIL
RferPro120_KAF7288075  .....................................LYFKRIN-------------------------------------K..................

                               610       620       630       640       650       660       670       680       690       700
                       ....|....|....|....|....|....|....|....|....|....|....|....|....|....|....|....|....|....|....|....|
RferPro120_KAF7288074  LVTLILDTESRSFISKVYPKFKQSQPEIGEFPGVPVILTPLIESGKIYDARLASQVQFNGFLGKTSFSGYFTVDKTYESNLFFWYFPCETGDKDAPLLLW
RferPro120_KAF7288075  ....................................................................................................

                               710       720       730       740       750       760       770       780       790       800
                       ....|....|....|....|....|....|....|....|....|....|....|....|....|....|....|....|....|....|....|....|
RferPro120_KAF7288074  LQGGPGATSLIGIFVENGPFETKFEHGLRERTTSWTKNHSVIYIDNPVGTGYSFTTGGYAQNQTIVGEHIYRALKQFFTVFPEEKKKDFYITGESYAGKY
RferPro120_KAF7288075  ....................................................................................................

                               810       820       830       840       850       860       870       880       890       900
                       ....|....|....|....|....|....|....|....|....|....|....|....|....|....|....|....|....|....|....|....|
RferPro120_KAF7288074  IPAVAYTIYQKNSGQNETINLKGLAIGDGFCDPINQLEYADYLYQIGLYDASTRDNIKNLEKQGLQMIQNKQFAEAARFFDSLMDGDFSNTTKFKNATGF
RferPro120_KAF7288075  ....................................................................................................

                               910       920       930       940       950       960       970       980       990      1000
                       ....|....|....|....|....|....|....|....|....|....|....|....|....|....|....|....|....|....|....|....|
RferPro120_KAF7288074  DNYDNFLYPIDPLNTGMELMAKYIVQDDVRAAIHVGNATFNEDSAVEENLLEDLMQSVTPWITELLKHYKILFYNGQLDIIVGYPLTVNFLNHLDFNAAT
RferPro120_KAF7288075  ....................................................................................................

                              1010      1020      1030      1040      1050      1060      1070      1080      1090      1100
                       ....|....|....|....|....|....|....|....|....|....|....|....|....|....|....|....|....|....|....|....|
RferPro120_KAF7288074  EYQTAARHQWIVDSNIAGYVKQAGNLTEILVRNAGHMVPADQPEWALDMITRFSHGSLVYITTNSKMNTVFFIFGLYIIKQSEGYYKDKPLPHRELEDVG
RferPro120_KAF7288075  ....................................................................................................

                              1110      1120      1130      1140      1150      1160      1170      1180      1190      1200
                       ....|....|....|....|....|....|....|....|....|....|....|....|....|....|....|....|....|....|....|....|
RferPro120_KAF7288074  EPLIVTPYIENGSINEAQQKAKVTYSELTKNNLTSYSGYFTVDKTYNSNLFFWFFLSENNPSEDPVILWLQGGPGASSLYGLFTENGPYEVNENLELSYR
RferPro120_KAF7288075  ....................................................................................................

                              1210      1220      1230      1240      1250      1260      1270      1280      1290      1300
                       ....|....|....|....|....|....|....|....|....|....|....|....|....|....|....|....|....|....|....|....|
RferPro120_KAF7288074  DYSWTRDHSVLFIDNPAGTGFSFTDSGFAQNETKVGDDLYSALVQFFTLFPDLQNNDFYVTGESYGGKYVPSISYTIHKNNPGADLKINLQGFAIGNPLS
RferPro120_KAF7288075  ....................................................................................................

                              1310      1320      1330      1340      1350      1360      1370      1380      1390      1400
                       ....|....|....|....|....|....|....|....|....|....|....|....|....|....|....|....|....|....|....|....|
RferPro120_KAF7288074  DPINQADYGSYMYYLGLIDSNLKTNLKSLHAMFTCAIRRQEYEKATNLLETIRSKMHNSNGLPNVYNHQAIEEEDPEYFATYLSQDELRPQVHVGNVEYG
RferPro120_KAF7288075  ....................................................................................................

                              1410      1420      1430      1440      1450      1460      1470      1480      1490      1500
                       ....|....|....|....|....|....|....|....|....|....|....|....|....|....|....|....|....|....|....|....|
RferPro120_KAF7288074  ISSDTVYDNLLADKSKSIAPWIEELLNNYRALFYNGQLDTADPYPLMVNFLKKLSFNGHDEYLAGERQIWRVGDEIAGYWKTGGYLTEVLVRDAGHMVPT
RferPro120_KAF7288075  ....................................................................................................

                              1510      1520
                       ....|....|....|....|
RferPro120_KAF7288074  FQPKWSYDLIYKFVRNTTLD
RferPro120_KAF7288075  ....................

                                10        20        30        40        50        60        70        80        90       100
                       ....|....|....|....|....|....|....|....|....|....|....|....|....|....|....|....|....|....|....|....|
RferPro123_KAF7273463  MDKSKFQIVLLTALIASYVSQECKDVKPDKFPYQAIVKSSVTCGGALISARHVVTLASCVYGSKEVKVFLGFYGYPYEQRNWNEPQAQICKSKQIIIHDS
RferPro123_KAF7273462  --MISLRTL.FFSFLTNSLAK...............................................................................

                               110       120       130       140       150       160       170       180       190       200
                       ....|....|....|....|....|....|....|....|....|....|....|....|....|....|....|....|....|....|....|....|
RferPro123_KAF7273463  FIKLSPTKYINNVAIAVLIQPARLTSAVQPIALSTEAPLPGSYLNVSGWTAKNNHKLRYCRASILNTDTCSYQFGSELLPSQEFCLQWYTSTRLNLIGNI
RferPro123_KAF7273462  ....................................................................................................

                               210       220       230       240       250       260       270       280       290       300
                       ....|....|....|....|....|....|....|....|....|....|....|....|....|....|....|....|....|....|....|....|
RferPro123_KAF7273463  ITANGQLVGFQSFTPKCLKTTKTCTGNDVITNILPFVRWIQASTGIPLRTPNTCSSNATVGQQQITQDYKDQLANINKELERLSSVQSRDTNEIFSKIKN
RferPro123_KAF7273462  ....................................................................................................
```

```
                              310       320       330       340       350       360       370       380       390       400
                          ....|....|....|....|....|....|....|....|....|....|....|....|....|....|....|....|....|....|....|....|
RferPro123_KAF7273463     VTEQCQQLSKGDVDVKYQKEISELRALIESNKVQLLLVEKTQNTTLDAINELKNDVSQQHNDSQALSNLIDAKCKECKTDMEKQVKLIEDLEKNNMRDVN
RferPro123_KAF7273462     ....................................................................................................

                              410       420       430       440       450       460       470       480
                          ....|....|....|....|....|....|....|....|....|....|....|....|....|....|....|....|
RferPro123_KAF7273463     EKLQFLSDEIVSLYKNLSNIKQEVDQKPASKDIVAINKQLIVEEVKKENQNILNHIASLNRTLQSLLQKLQTTIVFPELT
RferPro123_KAF7273462     ................................................................................

                               10        20        30        40        50        60        70        80        90       100
                          ....|....|....|....|....|....|....|....|....|....|....|....|....|....|....|....|....|....|....|....|
RferPro128_KAF7288074     MDNLDILRNTLLPTCLGIIPIIINYVLFTQQFYSTASELSKYAINLILDVINQQIFETFLNCLIIHRVFVILSSACSNTMKNLVFIFLAVVFYVNARVTY
RferPro128_KAF7288075     ....................................................................................................

                              110       120       130       140       150       160       170       180       190       200
                          ....|....|....|....|....|....|....|....|....|....|....|....|....|....|....|....|....|....|....|....|
RferPro128_KAF7288074     PNVYKPIQTLELTSDEDVGDPLILTPLLKENKIEEARQKSTVNITGFETVESYSGFFTVDENFDSNLFFWFFPSENDFQNDPVLLWLQGGPGASSLYALF
RferPro128_KAF7288075     ....................................................................................................

                              210       220       230       240       250       260       270       280       290       300
                          ....|....|....|....|....|....|....|....|....|....|....|....|....|....|....|....|....|....|....|....|
RferPro128_KAF7288074     CENGPLIVLNGDQLSLREYSWTKNHSVLYIDNPAGTGFSFTNGGYAQNETKVGEDLYQALVQFFTLFPEIAENPFFVTGESYAGKYIPAIGYAIYLHRNT
RferPro128_KAF7288075     ....................................................................................................

                              310       320       330       340       350       360       370       380       390       400
                          ....|....|....|....|....|....|....|....|....|....|....|....|....|....|....|....|....|....|....|....|
RferPro128_KAF7288074     EDIQINLKGLLIGDGLTDPENQISEYGNYLYELGFIDQNGKEEFIKYQNETIQYIQQNDFLKAFDTFDILLNGDFTSPTVFQNLTGFSSYFNYLYRPSPD
RferPro128_KAF7288075     ....................................................................................................

                              410       420       430       440       450       460       470       480       490       500
                          ....|....|....|....|....|....|....|....|....|....|....|....|....|....|....|....|....|....|....|....|
RferPro128_KAF7288074     DDNSYEAFLQSADVRKAIHVGNLSYSSENVEENLREDLLQSVAPWVAELLSNYRMLFFNGQLDIIVAYPLTINFLNNLEFSGSDEYKTAPRSIWTVDDEV
RferPro128_KAF7288075     ....................................................................................................

                              510       520       530       540       550       560       570       580       590       600
                          ....|....|....|....|....|....|....|....|....|....|....|....|....|....|....|....|....|....|....|....|
RferPro128_KAF7288074     AGYVKVAANLTEVLVRNSGHMVPMDQPKWAYDLVYKFFGSEKVQSRGEKRAVHKVQYIRKRTVNKRWWRNTKNKLKSITTENTVKSSTEKMFRFIFIAIL
RferPro128_KAF7288075     .....................................LYFKRIN-------------------------------------K..................

                              610       620       630       640       650       660       670       680       690       700
                          ....|....|....|....|....|....|....|....|....|....|....|....|....|....|....|....|....|....|....|....|
RferPro128_KAF7288074     LVTLILDTESRSFISKVYPKFKQSQPEIGEFPGVPVILTPLIESGKIYDARLASQVQFNGFLGKTSFSGYFTVDKTYESNLFFWYFPCETGDKDAPLLLW
RferPro128_KAF7288075     ....................................................................................................

                              710       720       730       740       750       760       770       780       790       800
                          ....|....|....|....|....|....|....|....|....|....|....|....|....|....|....|....|....|....|....|....|
RferPro128_KAF7288074     LQGGPGATSLIGIFVENGPFETKFEHGLRERTTSWTKNHSVIYIDNPVGTGYSFTTGGYAQNQTIVGEHIYRALKQFFTVFPEEKKKDFYITGESYAGKY
RferPro128_KAF7288075     ....................................................................................................

                              810       820       830       840       850       860       870       880       890       900
                          ....|....|....|....|....|....|....|....|....|....|....|....|....|....|....|....|....|....|....|....|
RferPro128_KAF7288074     IPAVAYTIYQKNSGQNETINLKGLAIGDGFCDPINQLEYADYLYQIGLYDASTRDNIKNLEKQGLQMIQNKQFAEAARFFDSLMDGDFSNTTKFKNATGF
RferPro128_KAF7288075     ....................................................................................................

                              910       920       930       940       950       960       970       980       990      1000
                          ....|....|....|....|....|....|....|....|....|....|....|....|....|....|....|....|....|....|....|....|
RferPro128_KAF7288074     DNYDNFLYPIDPLNTGMELMAKYIVQDDVRAAIHVGNATFNEDSAVEENLLEDLMQSVTPWITELLKHYKILFYNGQLDIIVGYPLTVNFLNHLDFNAAT
RferPro128_KAF7288075     ....................................................................................................

                             1010      1020      1030      1040      1050      1060      1070      1080      1090      1100
                          ....|....|....|....|....|....|....|....|....|....|....|....|....|....|....|....|....|....|....|....|
RferPro128_KAF7288074     EYQTAARHQWIVDSNIAGYVKQAGNLTEILVRNAGHMVPADQPEWALDMITRFSHGSLVYITTNSKMNTVFFIFGLYIIKQSEGYYKDKPLPHRELEDVG
RferPro128_KAF7288075     ....................................................................................................

                             1110      1120      1130      1140      1150      1160      1170      1180      1190      1200
                          ....|....|....|....|....|....|....|....|....|....|....|....|....|....|....|....|....|....|....|....|
RferPro128_KAF7288074     EPLIVTPYIENGSINEAQQKAKVTYSELTKNNLTSYSGYFTVDKTYNSNLFFWFFLSENNPSEDPVILWLQGGPGASSLYGLFTENGPYEVNENLELSYR
RferPro128_KAF7288075     ....................................................................................................

                             1210      1220      1230      1240      1250      1260      1270      1280      1290      1300
                          ....|....|....|....|....|....|....|....|....|....|....|....|....|....|....|....|....|....|....|....|
RferPro128_KAF7288074     DYSWTRDHSVLFIDNPAGTGFSFTDSGFAQNETKVGDDLYSALVQFFTLFPDLQNNDFYVTGESYGGKYVPSISYTIHKNNPGADLKINLQGFAIGNPLS
RferPro128_KAF7288075     ....................................................................................................

                             1310      1320      1330      1340      1350      1360      1370      1380      1390      1400
                          ....|....|....|....|....|....|....|....|....|....|....|....|....|....|....|....|....|....|....|....|
RferPro128_KAF7288074     DPINQADYGSYMYYLGLIDSNLKTNLKSLHAMFTCAIRRQEYEKATNLLETIRSKMHNSNGLPNVYNHQAIEEEDPEYFATYLSQDELRPQVHVGNVEYG
RferPro128_KAF7288075     ....................................................................................................

                             1410      1420      1430      1440      1450      1460      1470      1480      1490      1500
                          ....|....|....|....|....|....|....|....|....|....|....|....|....|....|....|....|....|....|....|....|
RferPro128_KAF7288074     ISSDTVYDNLLADKSKSIAPWIEELLNNYRALFYNGQLDTADPYPLMVNFLKKLSFNGHDEYLAGERQIWRVGDEIAGYWKTGGYLTEVLVRDAGHMVPT
RferPro128_KAF7288075     ....................................................................................................

                             1510      1520
                          ....|....|....|....|
RferPro128_KAF7288074     FQPKWSYDLIYKFVRNTTLD
RferPro128_KAF7288075     ....................

                               10        20        30        40        50        60        70        80        90       100
                          ....|....|....|....|....|....|....|....|....|....|....|....|....|....|....|....|....|....|....|....|
RferPro132_KAF7273207     MTVSVIIVVFWFFLAEILGVQSKATDVPGRTALDNFPLIDGHNDLPYKLKMIYNNQIEKYDFLKEWQENDEMKDCKSCHTDYVKLTKGKLGAQFWSAYIK
RferPro132_KAF7273206     ...............---..................................................................................
```

```
                             110       120       130       140       150       160       170       180       190       200
                         ....|....|....|....|....|....|....|....|....|....|....|....|....|....|....|....|....|....|....|....|
RferPro132_KAF7273207    CTNNTNPVSDTIEQIDLIKRLVDKYSGVLYWATKSDDIAEAFKQKKIASFIGVEGGHSIDNRLSVLRAYYELGVRYLTLTHFCNLDWADSTAADSTPDTP
RferPro132_KAF7273206    ....................................................................................................

                             210       220       230       240       250       260       270       280       290       300
                         ....|....|....|....|....|....|....|....|....|....|....|....|....|....|....|....|....|....|....|....|
RferPro132_KAF7273207    KKNLTEFGKIIIAEMNRLGMMVDLAHVSRNVMIDAIKASKAPVIFSHSSSRYIYDHVRNVNDDVLQLLKENDGIIMVNFFSGFIGGNNTIYDVINHINHI
RferPro132_KAF7273206    ....................................................................................................

                             310       320       330       340       350       360       370       380
                         ....|....|....|....|....|....|....|....|....|....|....|....|....|....|....|....|....|
RferPro132_KAF7273207    VEVIGVDHVGLGADYDGVHRLPKGLEDVSTYSDLFDLLKVLNPQIWTIENLEKLAGRNLVRVFRKVEQVKLQLKDEKPREDIINL
RferPro132_KAF7273206    .....................................................................................


                              10        20        30        40        50        60        70        80        90       100
                         ....|....|....|....|....|....|....|....|....|....|....|....|....|....|....|....|....|....|....|....|
RferPro140_KAF7279864    MWRCIAGVVFLVPLLTAASINKRSIDLIAAHGLISDTKLPGDVTPNSYTLKLHPYPDHGNFSGEISINATVQHQTKQVVLHKHPDLRINLLRVVQILPSD
RferPro140_KAF7279863    ....................................................................................................

                             110       120       130       140       150       160       170       180       190       200
                         ....|....|....|....|....|....|....|....|....|....|....|....|....|....|....|....|....|....|....|....|
RferPro140_KAF7279864    YESRTMSPNSNEKGYKVQMLKTTWEQTTVSIEDSRISVGSRKVNKQSMISIDMDTVMKTGAVLEIYIEFEGYMFNDTTEGLFRNSYIDPETKQKKWYVAT
RferPro140_KAF7279863    ....................................................................................................

                             210       220       230       240       250       260       270       280       290       300
                         ....|....|....|....|....|....|....|....|....|....|....|....|....|....|....|....|....|....|....|....|
RferPro140_KAF7279864    FMRPNLARNVFPCFDEPAYKVPFQVTIIRPKHMRAIFNMPLESSSDPSSYWVADRFQRTPPMTTFSFAMVISELSHISINVSDSE----DIKMRVWARPD
RferPro140_KAF7279863    .....................................................................................VYFP...........

                             310       320       330       340       350       360       370       380       390       400
                         ....|....|....|....|....|....|....|....|....|....|....|....|....|....|....|....|....|....|....|....|
RferPro140_KAF7279864    FIEALTNVTTKVSKSVKFLEDFWGSPYPLPELNIFALPNYQATKPADSWGLLMFKESELSSKGSWHLTQELVYQWLGALATPFWWSDAHINNALVRYITA
RferPro140_KAF7279863    ....................................................................................................

                             410       420       430       440       450       460       470       480       490       500
                         ....|....|....|....|....|....|....|....|....|....|....|....|....|....|....|....|....|....|....|....|
RferPro140_KAF7279864    YATLKIGENETFNNWPTTMLYSIYYEFSKRYPHGKNTAIKQDSSSAKTELVFRMLNYTLGETTFKHGMQRFMSDRQFKTFFGDDIWLSLTEQARFDKTLP
RferPro140_KAF7279863    ....................................................................................................

                             510       520       530       540       550       560       570       580       590       600
                         ....|....|....|....|....|....|....|....|....|....|....|....|....|....|....|....|....|....|....|....|
RferPro140_KAF7279864    EPITINEIAGSWITKDRLPVVTVTRNYNDNTAKIYQRVYLRERPHDVPDQEKFLWWIPVVLIRQDKMNFRNTTPLIWMMKEREIILQDMPAADTFIIINP
RferPro140_KAF7279863    ....................................................................................................

                             610       620       630       640       650       660       670       680       690       700
                         ....|....|....|....|....|....|....|....|....|....|....|....|....|....|....|....|....|....|....|....|
RferPro140_KAF7279864    EEIGPFPVNYDTENWNMLAEYLQTENRTRIPVYTRAKLLHDAWNLAYAGDLSFATALNMTLFLKNEREYLAWDPVFTLIDHIGKHIDSSAVHKKFQTYVR
RferPro140_KAF7279863    ....................................................................................................

                             710       720       730       740       750       760       770       780       790       800
                         ....|....|....|....|....|....|....|....|....|....|....|....|....|....|....|....|....|....|....|....|
RferPro140_KAF7279864    LLLTPLYQELGNEPQEGESEGRAHLRSSSKVFLCQTGYKPCIEEAQAAFKKWMDCKNPDEGNPVANQYICPVFKWGTKEEWEFGLQRIIRFPPSRKPSER
RferPro140_KAF7279863    ....................................................................................................

                             810       820       830       840       850       860       870       880       890       900
                         ....|....|....|....|....|....|....|....|....|....|....|....|....|....|....|....|....|....|....|....|
RferPro140_KAF7279864    TYLLKTLTGCPNDPSKIERLLNITVLEQNGNFTDNDIYLIFSMLTGSANGYTTLFNFLRKNWDTIKTRFEDKPMLWNSIITSATTVFKTQEGLDMVSKLY
RferPro140_KAF7279863    ....................................................................................................

                             910       920       930       940       950
                         ....|....|....|....|....|....|....|....|....|....|..
RferPro140_KAF7279864    LERRTEFGNADFVIEKALKNIREETRWSNDNLPVIEKWLDGYLRNNEDKIDC
RferPro140_KAF7279863    ....................................................

                              10        20        30        40        50        60        70        80        90       100
                         ....|....|....|....|....|....|....|....|....|....|....|....|....|....|....|....|....|....|....|....|
RferPro177_KAF7286207    MLLHTNVSNRYAKTLITSKVKNLNTKAQETTFSIVTPEKAFISGFTMEIDGKTYEAYVKEKEEAKKTYDEAVASGIGAAHVAVSARDSNRFTVSVNIEPQ
RferPro177_KAF7286206    ....................................................................................................

                             110       120       130       140       150       160       170       180       190       200
                         ....|....|....|....|....|....|....|....|....|....|....|....|....|....|....|....|....|....|....|....|
RferPro177_KAF7286207    SKATFYLRYEELLVRKTGKYELVLNINPGQPVKNLVVEVNIIESRPLKFVKVPSLRT----VNDTTAIVTFKPDVVRQKLLTSILGGKEDDGLSGQFIVQ
RferPro177_KAF7286206    .........................................................DILI.......................................

                             210       220       230       240       250       260       270       280       290       300
                         ....|....|....|....|....|....|....|....|....|....|....|....|....|....|....|....|....|....|....|....|
RferPro177_KAF7286207    YDVARDPLGGEVLVDGGYFVHFFAPEDLPSLNKQILFVLDTSGSMGGRKIQQLKEAMNSILDELKHVNVSYRETPSWRLVAFPEEEQSSQYPKILPDSFP
RferPro177_KAF7286206    ....................................................................................................

                             310       320       330       340       350       360       370       380       390       400
                         ....|....|....|....|....|....|....|....|....|....|....|....|....|....|....|....|....|....|....|....|
RferPro177_KAF7286207    ASAENIKKAKEVVEMLRSGGGTDIQKGLEVALKVVNKYFDPKGNQPIIVFLTDGQPTEGTPESITSTITDHNKYKVPIFALSFGSGADKAFLQKISLKND
RferPro177_KAF7286206    ....................................................................................................

                             410       420       430       440       450       460       470       480       490       500
                         ....|....|....|....|....|....|....|....|....|....|....|....|....|....|....|....|....|....|....|....|
RferPro177_KAF7286207    GFARHIYEAADASLQLRNFYQEISSPLLTNVTFKYVNKVKNVTRTYFPILFNGTELCAAGITDVGFQPTLIEALGRRGPITLEPKVYQSTGSLERLWAYL
RferPro177_KAF7286206    ....................................................................................................

                             510       520       530       540       550       560       570       580       590       600
                         ....|....|....|....|....|....|....|....|....|....|....|....|....|....|....|....|....|....|....|....|
RferPro177_KAF7286207    TVKQLLEARQVAKDKESITEKALAIAVKYSFVTDITSLIVVKPNATSALDLEDASEHPTFMNLNRSFLTSAGSSGYGPYRFGGYTRSRLTGFRRISKVTR
RferPro177_KAF7286206    ....................................................................................................

                             610       620       630       640       650       660       670       680       690
```

```
                      ....|....|....|....|....|....|....|....|....|....|....|....|....|....|....|....|....|....|....|....|.
RferPro177_KAF7286207 ISAAALPPTTQAPSWIQKVLNNNETISVNARNYTLGLNEQFGEGNECSNSVDNSTGHCTLIHECPKVHNYLTDLETYKKYFCEQKSYAGVCCPSTP
RferPro177_KAF7286206 ................................................................................................

                              10        20        30        40        50        60        70        80        90       100
                      ....|....|....|....|....|....|....|....|....|....|....|....|....|....|....|....|....|....|....|....|
RferPro193_KAF7279864 MWRCIAGVVFLVPLLTAASINKRSIDLIAAHGLISDTKLPGDVTPNSYTLKLHPYPDHGNFSGEISINATVQHQTKQVVLHKHPDLRINLLRVVQILPSD
RferPro193_KAF7279863 ....................................................................................................

                             110       120       130       140       150       160       170       180       190       200
                      ....|....|....|....|....|....|....|....|....|....|....|....|....|....|....|....|....|....|....|....|
RferPro193_KAF7279864 YESRTMSPNSNEKGYKVQMLKTTWEQTTVSIEDSRISVGSRKVNKQSMISIDMDTVMKTGAVLEIYIEFEGYMFNDTTEGLFRNSYIDPETKQKKWYVAT
RferPro193_KAF7279863 ....................................................................................................

                             210       220       230       240       250       260       270       280       290       300
                      ....|....|....|....|....|....|....|....|....|....|....|....|....|....|....|....|....|....|....|....|
RferPro193_KAF7279864 FMRPNLARNVFPCFDEPAYKVPFQVTIIRPKHMRAIFNMPLESSSDPSSYWVADRFQRTPPMTTFSFAMVISELSHISINVSDSE----DIKMRVWARPD
RferPro193_KAF7279863 .....................................................................................VYFP...........

                             310       320       330       340       350       360       370       380       390       400
                      ....|....|....|....|....|....|....|....|....|....|....|....|....|....|....|....|....|....|....|....|
RferPro193_KAF7279864 FIEALTNVTTKVSKSVKFLEDFWGSPYPLPELNIFALPNYQATKPADSWGLLMFKESELSSKGSWHLTQELVYQWLGALATPFWWSDAHINNALVRYITA
RferPro193_KAF7279863 ....................................................................................................

                             410       420       430       440       450       460       470       480       490       500
                      ....|....|....|....|....|....|....|....|....|....|....|....|....|....|....|....|....|....|....|....|
RferPro193_KAF7279864 YATLKIGENETFNNWPTTMLYSIYYEFSKRYPHGKNTAIKQDSSSAKTELVFRMLNYTLGETTFKHGMQRFMSDRQFKTFFGDDIWLSLTEQARFDKTLP
RferPro193_KAF7279863 ....................................................................................................

                             510       520       530       540       550       560       570       580       590       600
                      ....|....|....|....|....|....|....|....|....|....|....|....|....|....|....|....|....|....|....|....|
RferPro193_KAF7279864 EPITINEIAGSWITKDRLPVVTVTRNYNDNTAKIYQRVYLRERPHDVPDQEKFLWWIPVVLIRQDKMNFRNTTPLIWMMKEREIILQDMPAADTFIIINP
RferPro193_KAF7279863 ....................................................................................................

                             610       620       630       640       650       660       670       680       690       700
                      ....|....|....|....|....|....|....|....|....|....|....|....|....|....|....|....|....|....|....|....|
RferPro193_KAF7279864 EEIGPFPVNYDTENWNMLAEYLQTENRTRIPVYTRAKLLHDAWNLAYAGDLSFATALNMTLFLKNEREYLAWDPVFTLIDHIGKHIDSSAVHKKFQTYVR
RferPro193_KAF7279863 ....................................................................................................

                             710       720       730       740       750       760       770       780       790       800
                      ....|....|....|....|....|....|....|....|....|....|....|....|....|....|....|....|....|....|....|....|
RferPro193_KAF7279864 LLLTPLYQELGNEPQEGESEGRAHLRSSSKVFLCQTGYKPCIEEAQAAFKKWMDCKNPDEGNPVANQYICPVFKWGTKEEWEFGLQRIIRFPPSRKPSER
RferPro193_KAF7279863 ....................................................................................................

                             810       820       830       840       850       860       870       880       890       900
                      ....|....|....|....|....|....|....|....|....|....|....|....|....|....|....|....|....|....|....|....|
RferPro193_KAF7279864 TYLLKTLTGCPNDPSKIERLLNITVLEQNGNFTDNDIYLIFSMLTGSANGYTTLFNFLRKNWDTIKTRFEDKPMLWNSIITSATTVFKTQEGLDMVSKLY
RferPro193_KAF7279863 ....................................................................................................

                             910       920       930       940       950
                      ....|....|....|....|....|....|....|....|....|....|..
RferPro193_KAF7279864 LERRTEFGNADFVIEKALKNIREETRWSNDNLPVIEKWLDGYLRNNEDKIDC
RferPro193_KAF7279863 ....................................................
```
