# Supplementary figures and images for: Genome-Wide Identification and Expression Profiling of Glycosidases, Lipases, and Proteases from Invasive Asian Palm Weevil, Rhynchophorus ferrugineus
Source: Insects. 2025 Apr 17;16(4):421. doi: 10.3390/insects16040421 (PMC12027728; doi:10.3390/insects16040421)

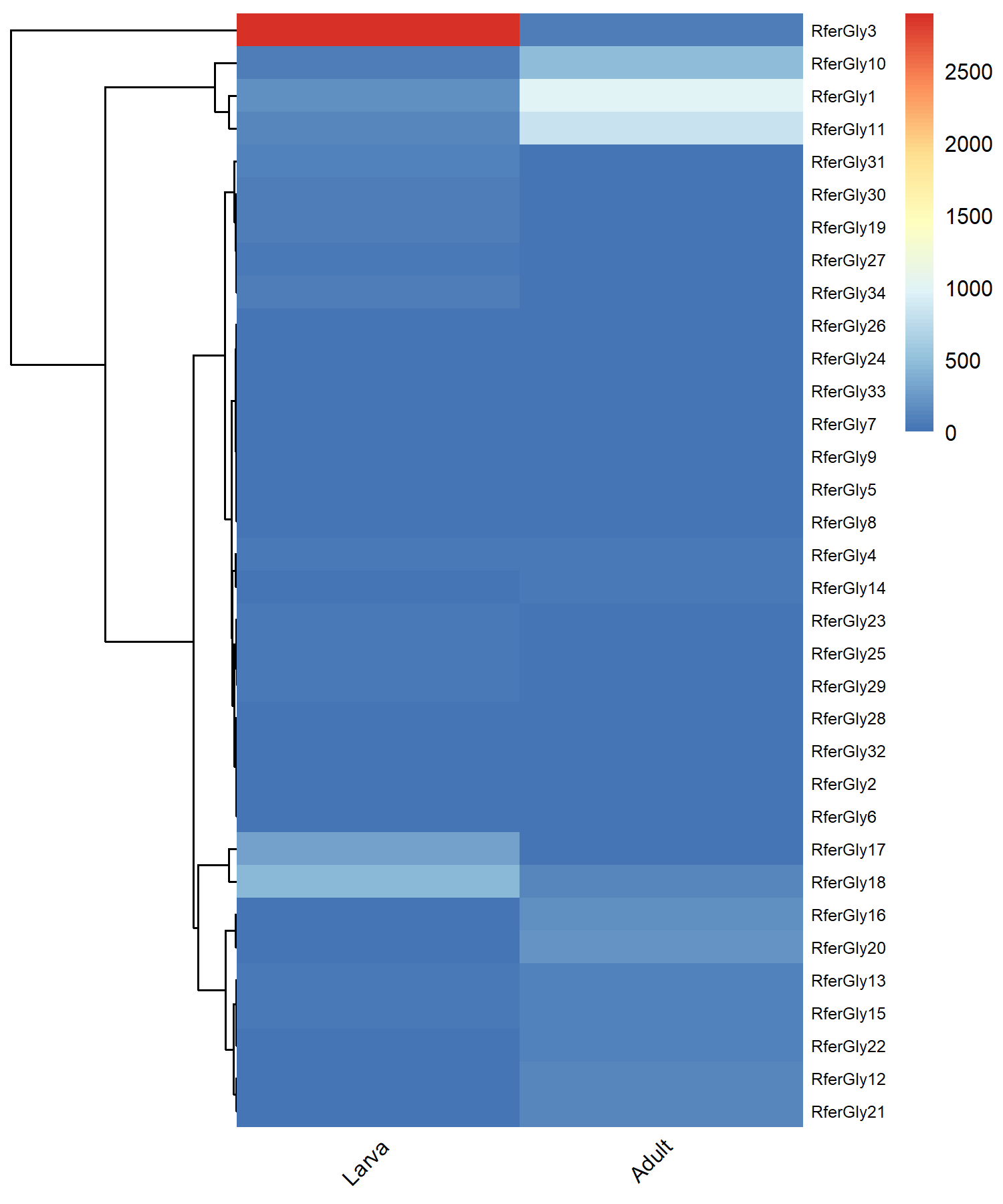

Supplement: Supplementary file 1 [file insects-16-00421-s001.zip › 080425_Supplementary Figures and Tables/Figure S1 - rfer_gly heatmap_300dpi.tiff]

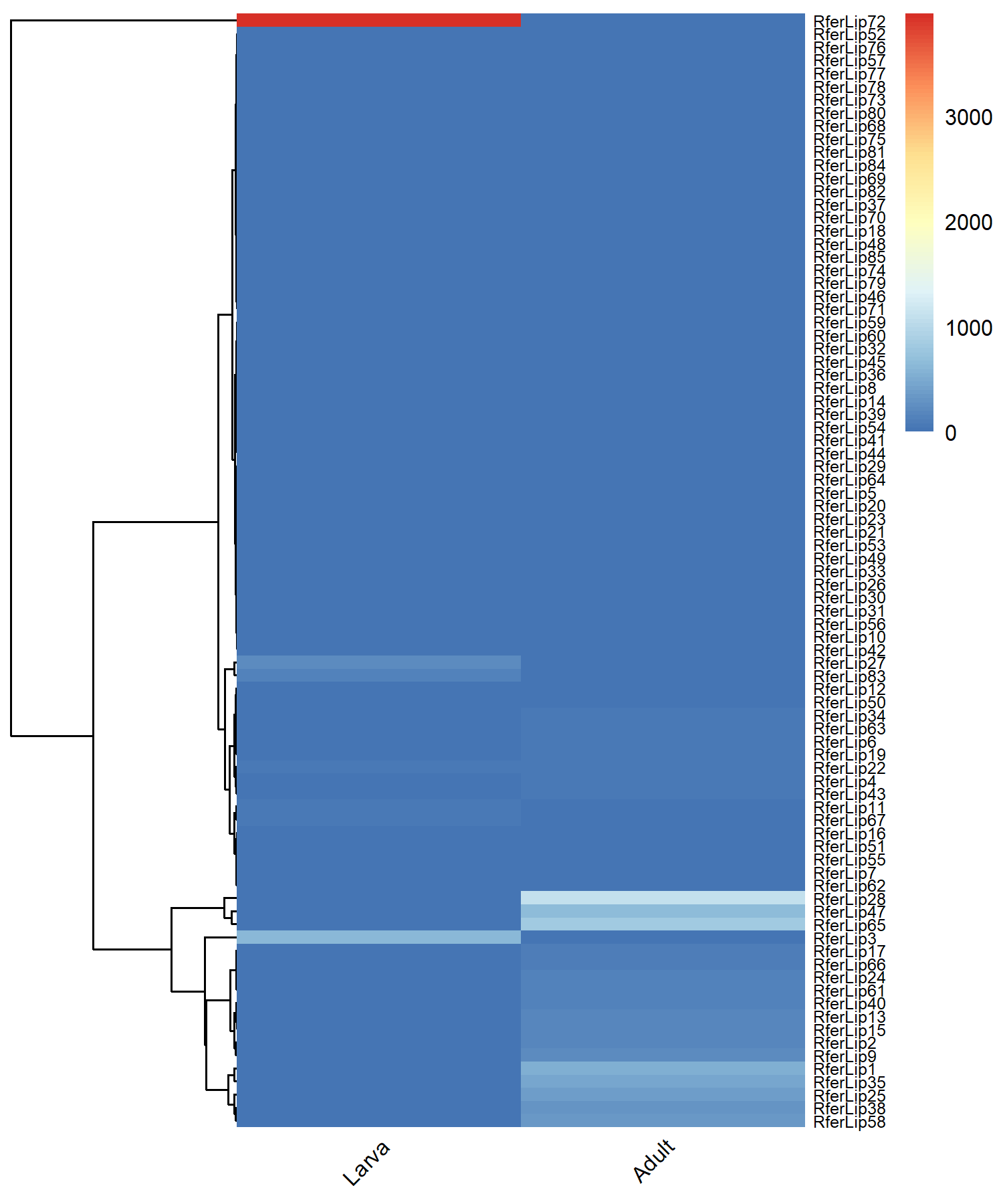

Supplement: Supplementary file 1 [file insects-16-00421-s001.zip › 080425_Supplementary Figures and Tables/Figure S2 - rfer_lip heatmap_300dpi.tiff]

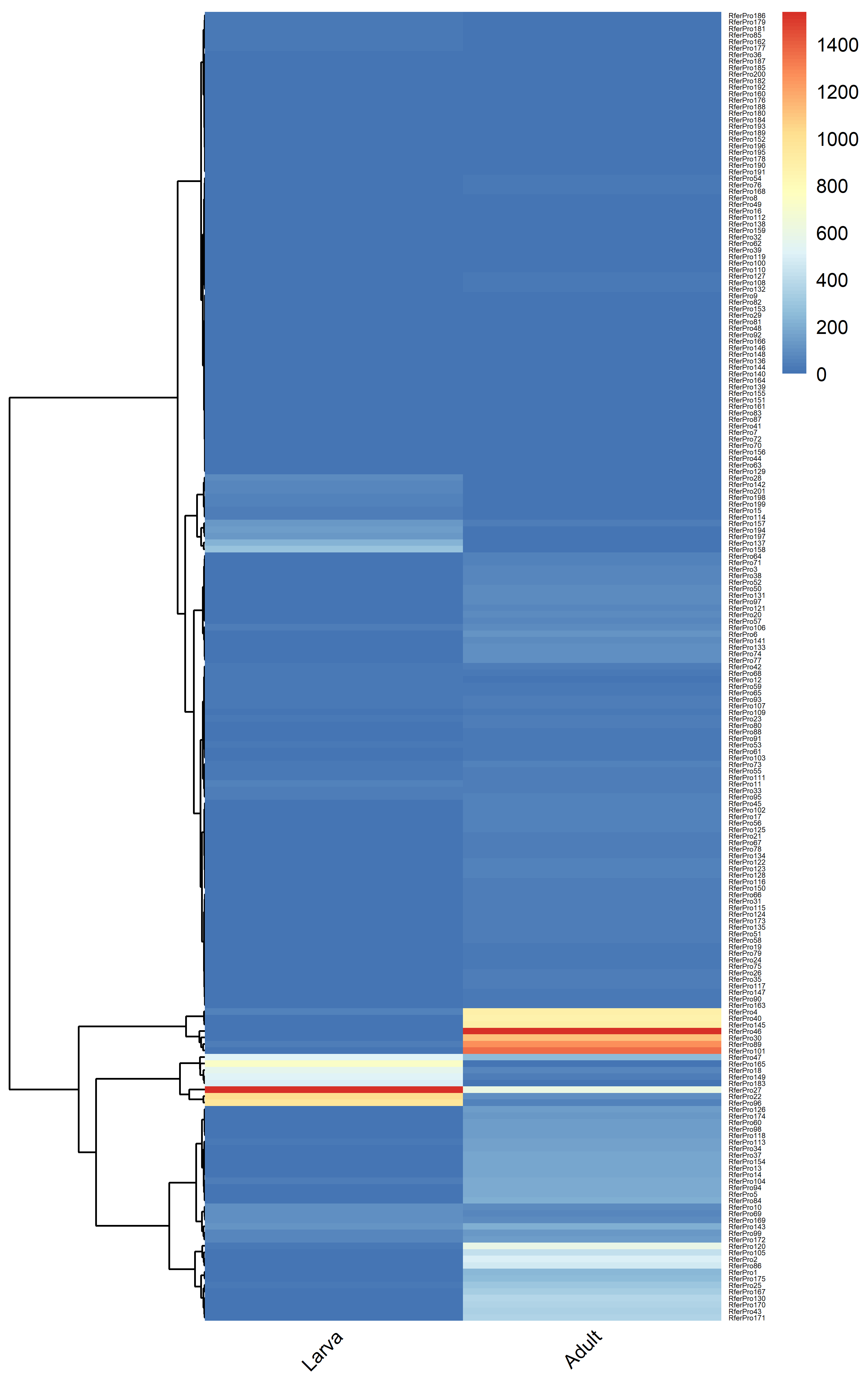

Supplement: Supplementary file 1 [file insects-16-00421-s001.zip › 080425_Supplementary Figures and Tables/Figure S3 - rfer_pro_heatmap_300dpi.tiff]
